# Design, Synthesis, *In Silico* Absorption, Distribution, Metabolism, and Elimination and Molecular Docking Studies of Thiazole‐Based Furan Derivatives, and Their Biological Evaluation for Alzheimer Disease Therapy

**DOI:** 10.1002/open.202500305

**Published:** 2025-08-10

**Authors:** Abdüllatif Karakaya, Ulviye Acar Çevik, Betül Kaya, Bilge Çiftçi, Adem Necip, Mesut Işık, Şükrü Beydemir, Yusuf Özkay, Zafer Asım Kaplancıklı

**Affiliations:** ^1^ Department of Pharmaceutical Chemistry Faculty of Pharmacy Zonguldak Bulent Ecevit University Zonguldak 67600 Turkey; ^2^ Institute of Graduate Education Anadolu University Eskişehir 26470 Turkey; ^3^ Department of Pharmaceutical Chemistry Faculty of Pharmacy Anadolu University Eskişehir 26470 Turkey; ^4^ Vocational School of Health Services Pharmacy Services Bilecik Şeyh Edebali University Bilecik 11230 Turkey; ^5^ Vocational School of Health Services Bilecik Şeyh Edebali University Bilecik 11230 Turkey; ^6^ Department of Pharmacy Services Vocational School of Health Services Harran University Şanlıurfa 63300 Turkey; ^7^ Department of Bioengineering Faculty of Engineering Bilecik Şeyh Edebali University Bilecik 11230 Turkey; ^8^ Department of Biochemistry Faculty of Pharmacy Anadolu University Eskişehir 26470 Turkey; ^9^ The Rectorate of Bilecik Şeyh Edebali University Bilecik 11230 Turkey

**Keywords:** Alzheimer's disease, cholinesterase inhibitors, furan, molecular docking, thiazole

## Abstract

Herein, a series of novel 5‐hydroxymethylfuran incorporated thiazole‐based furan derivatives are synthesized and characterized. The in vitro inhibitory potentials of the derivatives against acetylcholinesterase (AChE) and butyrylcholinesterase (BChE) are evaluated. In addition, the inhibitory potential of the thiazole‐based furan derivatives against AChE (4EY7) and BChE (4BDS) proteins is examined as *in silico*. For this purpose, the effects of the compounds on human metabolism are evaluated with absorption, distribution, metabolism, excretion, and toxicity programming. Furthermore, their antioxidant potential is assessed through 1,1‐diphenyl‐2‐picrylhydrazyl (DPPH) and 2,2′‐azino‐bis(3‐ethylbenzthiazoline‐6‐sulfonic acid) (ABTS) radical scavenging assays. The enzymatic inhibition studies reveal that all compounds exhibit inhibitory effects on both AChE and BChE. Among them, compound 2b demonstrates the most potent inhibition against AChE, with a *K*_I_ value of 14.887 ± 1.054 μM, whereas compound 2f exhibits the highest inhibitory activity against BChE, with a *K*_I_ value of 4.763 ± 0.321 μM. Compounds 2a (12.202% for DPPH and 56.842% for ABTS) and 2i (13.309% for DPPH and 31.842% for ABTS) are among the most active compounds for both radical scavenging tests. These findings highlight that the synthesized derivatives possess promising dual cholinesterase (ChE) inhibitory activity as well as radical scavenging potential. These activities emphasize their potential as therapeutic candidates for neurodegenerative disorders such as Alzheimer's disease.

## Graphical abstract

**GA:** Oxidative stress plays a critical role in Alzheimer's disease (AD) by promoting A*β* plaque accumulation and tau pathology, accelerating neurodegeneration. Elevated reactive oxygen species contribute to cognitive decline through extensive oxidative damage. Therapeutic strategies targeting this mechanism include cholinesterase inhibitors, which enhance cholinergic neurotransmission, and antioxidant agents, which reduce oxidative stress. Combined, these approaches may slow disease progression and offer symptomatic and neuroprotective benefits in AD treatment.

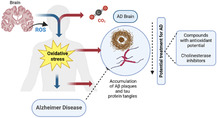

## 1 Introduction

Alzheimer's disease (AD) is a progressive and irreversible neurodegenerative disorder characterized by a decline in memory and other cognitive functions, significantly impacting patients’ social lives.^[^ ^1^ ^]^ AD represents the most prevalent cause of dementia, particularly among the elderly, accounting for approximately 60% to 70% of cases.^[^ ^2^ ^]^ The etiology of AD is complex and multifactorial, involving cholinergic deficiency, the accumulation of amyloid plaques, and neurofibrillary tangles composed of aggregated tau protein within the brain.^[^ ^3^ ^]^

While the precise pathogenic mechanisms underlying AD remain incompletely elucidated, the multifactorial nature of the disease, arising from a complex interplay of neurochemical factors, is widely acknowledged within the scientific community. Current understanding of AD pathogenesis encompasses cholinergic dysfunction,^[^ ^4^ ^]^ oxidative stress,^[^ ^5^ ^]^ mitochondrial dysfunction,^[^ ^6^ ^]^ metal dyshomeostasis,^[^ ^7^ ^]^ *β*‐amyloid (A*β*) oligomerization,^[^ ^8^ ^]^ the cell cycle,^[^ ^9^ ^]^ *τ*‐protein hyperphosphorylation, and aggregation^[^ ^10^ ^]^ hypothesis. This evolving understanding of AD pathogenesis has spurred the development of numerous therapeutic strategies, leading to the discovery and development of a range of agents, including both small synthetic compounds and macromolecules. Numerous compounds are currently undergoing pre‐clinical or clinical investigation; however, the failure rate in AD drug discovery remains exceedingly high. The majority of current AD treatments are predicated on the cholinergic hypothesis, which posits that dysregulation of the cholinergic system, primarily due to declining acetylcholine (ACh) levels, underlies this cognitive disorder. Consequently, restoring ACh levels has become a primary therapeutic strategy in AD therapeutics.^[^ ^11^ ^]^ It is well established that two cholinesterase (ChE) enzymes, acetylcholinesterase (AChE) and butyrylcholinesterase (BChE), are responsible for the hydrolysis of ACh within the human brain. Therefore, ChE inhibitors are used to elevate ACh concentrations in the brains of AD patients, thereby potentially providing therapeutic benefit.^[^ ^12^ ^]^ Prior research suggests that AChE exhibits greater substrate specificity than BChE in the human brain,^[^ ^13^ ^]^ leading to the prevailing view that AChE inhibitors may offer more targeted therapeutic effects compared to BChE inhibitors. While numerous potential drug targets for AD treatment are currently under investigation, AChE inhibitors remain the primary therapeutic agents in clinical practice. Notably, in the later stages of AD, the critical role of BChE has highlighted in late‐stage AD patients, who experience progressive AChE loss.^[^ ^11^ ^]^ Consequently, the development of selective BChE inhibitors is gaining increasing attention from drug developers. Currently, three ChE inhibitors are used clinically: donepezil and galantamine, which are selective AChE inhibitors, and rivastigmine, a dual AChE‐BChE inhibitor.^[^ ^10^ ^,^ ^14^ ^]^

The active site of human AChE is characterized by a ≈20 Å long gorge, featuring two crucial regions: the catalytic active site (CAS) located at the gorge's base, and the peripheral anionic site (PAS) situated near the entrance. These two sites are interconnected by a narrow groove. The CAS facilitates ACh hydrolysis via a catalytic triad composed of Ser200, Glu327, and His440. The PAS, comprised of aromatic residues such as Tyr70, Tyr121, and Trp279, plays a significant role in both ACh hydrolysis and amyloid‐beta (A*β*) aggregation. Consequently, compounds capable of interacting with both the CAS and PAS are hypothesized to exhibit multiple therapeutic benefits, driving the development of multitarget‐directed ligands. While the overall shape and organization of the BChE active site resemble those of AChE, the BChE catalytic site possesses a considerably larger volume. This structural difference presents a target for the design of selective BChE inhibitors.^[^ ^10^ ^,^ ^15^ ^,^ ^16^ ^]^ The current repertoire of chemical scaffolds for approved ChEIs is limited, and existing market offerings provide only palliative relief, failing to address the underlying neurodegenerative processes.^[^ ^17^ ^]^ Consequently, the development of ChEIs based on novel scaffolds remains a critical objective in drug discovery. This study details our computational approach to identifying new ChE inhibitors. Utilizing donepezil, a highly active AChE inhibitors, as a template, we employed a workflow integrating shape‐based comparison, structure‐based pharmacophore‐mediated virtual screening, and molecular docking. This methodology facilitated the identification of some novel compounds demonstrating significant inhibitory activity against ChEs.

Oxidative stress is also reported to be a crucial contributor in the initiation and progression of AD. Oxidative stress is caused by the excess upregulation of reactive oxygen species (ROS) such as superoxide (O^2•−^), hydrogen peroxide (H_2_O_2_), peroxynitrite (ONOO^−^), and hydroxyl radicals (HO^•^).^[^ ^18^ ^,^ ^19^ ^]^ Aberrantly high ROS levels contribute to neurodegeneration through the disruption of biomolecules including lipids, proteins, and nucleic acids of the cells.^[^ ^20^ ^]^ Oxidative damage to DNA can also result in the accumulation of A*β* plaques and tau protein tangles. Antioxidant therapy appears to be helpful with respect to reducing DNA oxidation and decelerating the progression of AD.^[^ ^21^ ^]^

In AD, an increase in the ROS leads to elevated oxidative stress, resulting in significant oxidative damage. This oxidative stress has been shown to promote the accumulation of A*β* plaques and the formation of tau protein tangles, which accelerate neurodegeneration and contribute to cognitive decline. Therefore, cholinesterase inhibitors and antioxidant agents are considered crucial therapeutic strategies in the treatment of AD. The ChE inhibitors enhance neurotransmission by increasing acetylcholine levels, while antioxidant agents mitigate oxidative stress, thereby potentially slowing down neurodegenerative processes. Consequently, the development of compounds that exhibit both enzyme inhibition and antioxidant properties represents a promising approach for delaying disease progression.

Thiazole is a versatile and valuable skeleton in the design of novel compounds of medicinal importance as anti‐Alzheimer's agents.^[^ ^22^, ^24^, ^25^ ^–^ ^26^ ^]^ The commercially available thiazole‐containing drugs highlighted the wide range of applications of thiazole scaffolds in medicinal chemistry. Both an electron‐donating group (–S–) and an electron‐accepting group (C=N) are present in thiazole ring, resulting in a stable heterocyclic compound.^[^ ^27^ ^,^ ^28^ ^]^ The remarkable efficiency of the thiazole core in varied interactions with prominent pharmacological targets extends the scope of drug design and discovery. In this context, the present study was designed to further explore the therapeutic promise of the thiazole moiety by synthesizing a novel compound featuring a structurally distinct thiazole analog. By integrating this scaffold into a new molecular framework, our aim was to investigate whether such modification could yield comparable or superior inhibitory effects on AChE and BChE. The supportive evidence from prior studies not only validates the relevance of our synthetic strategy but also reinforces the hypothesis that diversification of thiazole analogs may lead to new candidates with enhanced efficacy and favorable pharmacological profiles for the treatment of AD.

This study employed molecular docking simulations using an in‐house chemical library to identify compounds with high binding affinity and selectivity for AChE and BChE. Docking analysis indicated that some of the previously synthesized compounds by our research group^[^ ^23^ ^,^ ^29^, ^30^ ^–^ ^31^ ^]^ exhibited a favorable binding mode within the AChE/ BChE active site, closely resembling the binding orientation of the known cholinesterase inhibitor, donepezil. Based on the structural features of these compounds, in this paper, novel compounds were synthesized for subsequent biological evaluation. The aim of this study was to synthesize new 5‐hydroxymethylfuran incorporated thiazole‐based furan derivatives with radical scavenging and inhibitory potential for cholinesterase enzymes. The ABTS and DPPH radical scavenging potential of the synthesized derivatives and their inhibitory potential against cholinesterase enzymes were determined. In addition, the inhibitory potential of the synthesized thiazole‐based furan derivatives against AChE (4EY7) and BChE (4BDS) proteins was examined. For this purpose, the applied addition processes were performed. Then, the effects of the compounds on human metabolism were evaluated. For this, absorption, distribution, metabolism, excretion, and toxicity (ADME/T) programming is performed.

## 2 Results and Discussion

### 2.1 Chemistry

**Scheme 1:**
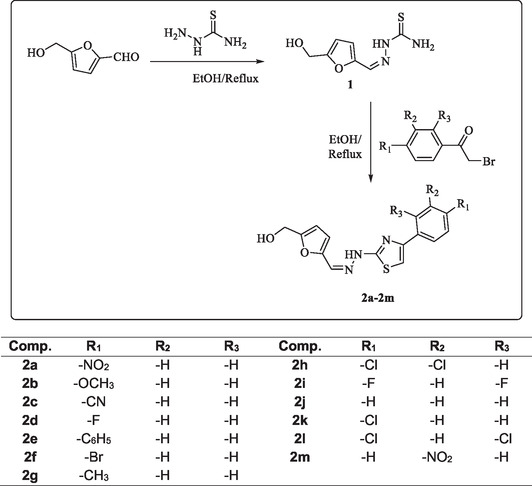
Chemical structure and general procedure for the synthesis of the final compounds **2a‐2 m.**

The general structures and synthetic procedure of title furan‐based thiazole derivatives **2a‐2m** synthesized in this study are shown in **Scheme** **1**. 5‐(Hydroxymethyl)furan‐2‐carbaldehyde was treated to thiosemicarbazide in the first step to afford thiosemicarbazone derivative which then reacted with the appropriate 2‐bromoacetophenones to synthesized compounds (**2a‐2m**).

The structures of all compounds were elucidated through ^1^H NMR and ^13^C NMR, and their structural characterization results are presented as Supplementary File (Figure S1‐30, Supporting Information).

### 2.2 Biological Activity

The role of oxidative stress in the pathogenesis of metabolic disorders is important. It is known in the literature that oxidative stress plays a role in the pathogenesis of Alzheimer's and many diseases.^[^ ^32^, ^33^ ^–^ ^34^ ^]^ In this study, a comprehensive study was conducted to evaluate the antioxidant potential of newly synthesized thiazole‐based furan derivatives (**2a‐2m**), which is an important indicator in reducing oxidative stress. The investigation primarily focused on their efficacy in scavenging DPPH^·^ and ABTS^+·^ radicals, which are widely accepted methods for assessing antioxidant activity. To ensure a robust comparative analysis, these derivatives were evaluated alongside trolox, used as a standard antioxidant reference. The findings, summarized in **Table** **1**, provided significant insights into their potential antioxidant efficacy.

**Table 1:** The radical removal potential of thiazole‐based furan derivatives (**2a‐2m**).

Compounds	DPPH[a] (2 µg mL^−1^)	ABTS[a] (2 µg mL^−1^)
**2a**	12.202 ± 0.965	56.842 ± 4.346
**2b**	1.109 ± 0.762	2.105 ± 0.176
**2c**	3.512 ± 0.237	15.789 ± 1.365
**2d**	1.848 ± 0.095	6.316 ± 0.584
**2e**	4.621 ± 0.372	17.105 ± 1.248
**2f**	7.579 ± 0.648	22.107 ± 1.728
**2g**	4.067 ± 0.289	11.053 ± 1.012
**2hr**	5.361 ± 0.486	12.106 ± 1.036
**2i**	13.309 ± 1.278	31.842 ± 2.785
**2j**	9.057 ± 0.834	28.158 ± 2.075
**2k**	6.470 ± 0.574	11.842 ± 0.982
**2l**	4.436 ± 0.386	21.053 ± 1.793
**2m**	11.275 ± 1.021	30.789 ± 2.489
Trolox	13.863 ± 1.023	29.211 ± 2.043

[a] Values are expressed as percent radical scavenging particles at 2 µg mL^−1^. Standard antioxidants (trolox).

In this study, the radical scavenging potential of the synthesized compounds (**2a‐2m**) was systematically assessed using ABTS^•+^ and DPPH^•^ scavenging assays. The results demonstrated that the compounds exhibited ABTS^•+^ scavenging activity ranging from 2.105% to 56.842% at a concentration of 2 μg mL^−1^, while their DPPH^•^ scavenging activity varied between 1.109% and 13.309% at a concentration of 2 μg mL^−1^. Notably, as detailed in Table 1, compounds **2a** and **2i** exhibited a radical scavenging effect in the DPPH assay, whereas compounds **2a**, **2i,** and **2m** displayed significant ABTS^•+^ scavenging activity, comparable to certain standard antioxidant trolox (13.863 ± 1.023 for DPPH and 29.211 ± 2.043 for ABTS). Compounds **2a** and **2i** are among the most active compounds for both radical scavenging tests with comparable potency to the standard compound. According to results, it can be assumed that *para*‐nitro (compound **2a**), *orto, para*‐difluoro (compound **2i**) substituents have positive contribution on DPPH^•^ scavenging activity, whereas both these substituents and *meta*‐nitro group (compound **2m**) increase the ABTS^•+^ scavenging activity.

These findings highlight the potential of these novel derivatives as antioxidant agents, with some compounds demonstrating promising radical scavenging efficacy, particularly in the ABTS assay. Further structural analysis and mechanistic studies are warranted to elucidate the relationship between their molecular framework and antioxidant activity.

This study explores the inhibitory potential of thiazole‐based furan derivatives in regulating the catalytic activities of AChE and BChE, which serve as key therapeutic targets in the treatment of AD. The primary objective is to synthesize and characterize selective derivatives with high affinity for ChE enzymes, thereby assessing their potential as effective enzyme inhibitors. Additionally, the study aims to identify novel therapeutic candidates for AD by modulating cholinesterase activity through enzymatic inhibition. A comprehensive analysis of the inhibitory properties of these derivatives, including their enzyme selectivity and potency, is presented in **Table** **2**, providing valuable insights into their pharmacological relevance and potential application in AD therapy.

**Table 2:** Kinetic parameters (IC50 and KI values) and docking score as inhibitory potential of thiazole‐based furan derivatives (**2a‐2m**) against AChE and BChE.

Compound ID	AChE[a]					BChE[b]					AChE/BChE
	*IC*_50_ [μM]	*R* ^2^	*K*_*I*_ [μM]	*R* ^2^	Docking score [kcal/mol]	*IC*_50_ [μM]	*R* ^2^	*K*_*I*_ [μM]	*R* ^2^	Docking score [kcal/mol]	SI *
**2a**	21.656	0.939	**14.887± 1.054**	0.978	−8.777	8.772	0.935	6.029 ± 0.523	0.967	−6.519	2.469
**2b**	21.105	0.956	**14.511 ± 1.122**	0.968	−8.790	34.652	0.951	23.823 ± 0.132	0.961	−5.728	0.609
**2c**	46.201	0.946	31.766 ± 2.856	0.974	−8.893	13.861	0.978	9.528 ± 0.824	0.948	−5.377	3.334
**2d**	53.308	0.953	36.656 ± 3.217	0.975	−8.674	10.828	0.964	7.442 ± 0.565	0.946	−7.637	4.925
**2e**	49.502	0.979	34.033 ± 2.105	0.972	−8.926	9.365	0.986	6.436 ± 0.452	0.939	−4.554	5.287
**2f**	38.501	0.948	26.469 ± 1.805	0.968	−8.697	6.932	0.962	**4.763 ± 0.321**	0.962	−7.135	5.557
**2g**	53.308	0.939	36.651 ± 2.564	0.975	−8.330	18.729	0.932	12.875 ± 1.208	0.948	−5.635	2.847
**2hr**	38.907	0.974	26.748 ± 2.043	0.978	−8.791	11.948	0.961	8.213 ± 0.722	0.956	−7.398	3.257
**2i**	63.102	0.949	43.386 ± 3.564	0.982	−9.181	9.493	0.957	6.524 ± 0.428	0.947	−7.596	6.650
**2j**	61.875	0.939	42.541 ± 3.486	0.975	−8.151	19.743	0.985	13.572 ± 1.048	0.934	−7.837	3.134
**2k**	57.277	0.964	39.379 ± 3.246	0.986	−8.739	12.375	0.962	8.506 ± 0.684	0.943	−7.294	4.629
**2l**	61.305	0.957	42.149 ± 3.793	0.982	−8.968	8.349	0.989	**5.738 ± 0.384**	0.934	−7.732	7.345
**2m**	86.625	0.977	59.558 ± 4.823	0.986	−9.131	21.656	0.978	14.888 ± 1.348	0.957	−7.131	4.001
**Tacrine**	0.165	0.986	0.058 ± 0.003	0.964	−9.473	0.215	0.948	0.063 ± 0.002	0.967	−8.787	0.920

[a] Acetylcholinesterase;.

[b] Butyrylcholinesterase;.

* SI (selectivity index)= *K*_I_ (AChE)/ *K*_I_ (BChE). The best results of *K*_I_ values are indicated in bold.

Previous studies have demonstrated the potential of various synthetic scaffolds as dual inhibitors of cholinesterase enzymes, thereby supporting the rationale of targeting both AChE and BChE in AD therapy. For instance, Hussain et al. (2022) synthesized a series of 24 benzimidazole‐based thiazole derivatives, all of which exhibited considerable inhibitory effects against AChE and BChE, with IC_50_ values ranging between 0.10 ± 0.05 and 11.10 ± 0.30 µM for AChE, and 0.20 ± 0.05 to 14.20 ± 0.10 µM for BChE.^[^ ^35^ ^]^ Similarly, triazole‐based thiosemicarbazone derivatives (6a–u) were reported to show moderate to strong cholinesterase inhibition, except for compounds 6c and 6d, which were inactive. The potent derivatives achieved IC_50_ values ranging between 0.10 ± 0.050 and 12.20 ± 0.30 µM for AChE and 0.20 ± 0.10 and 14.10 ± 0.40 µM for BChE.^[^ ^36^ ^]^ In another study, newly designed thiazole‐bearing sulfonamide analogs displayed promising anti‐Alzheimer activity, with compound 1 showing excellent potency with IC_50_ values ranging between 0.10 ± 0.05 and 11.40 ± 0.20 µM for AChE and 0.20 ± 0.050 and 14.30 ± 0.30 µM for BChE).^[^ ^37^ ^]^ These studies provide compelling evidence that structural modifications on the thiazole core can significantly enhance inhibitory potency and selectivity toward cholinesterase enzymes.

The synthesized thiazole‐based furan derivatives exhibited micromolar inhibitory activity against AD‐related ChE enzymes, with IC_50_ values ranging from 21.105 to 86.625 μM and *K*_I_ values between 14.511 ± 1.122 and 59.558 ± 4.823 μM for AChE. For BChE, IC_50_ values ranged from 6.932 to 34.652 μM, while *K*_I_ values were measured between 4.763 ± 0.321 and 23.823 ± 0.132 μM. Among the derivatives that were assessed, compounds **2a** with *para*‐nitro substituent and **2b** with *para*‐methoxy substituent exhibited notably higher inhibitory potency against AChE, with *K*_I_ values of 14.887 ± 1.054 μM and 14.511 ± 1.122 μM, respectively. These values indicate that it has a moderate AChE inhibitory potential, although it has a lower inhibitory potential than the reference inhibitor tacrine (IC_50_: 0.058 ± 0.003 μM). Enzyme kinetics analysis further revealed that compounds **2a** and **2b** displayed the highest selectivity for AChE, whereas compound **2m** exhibited lower selectivity, as indicated by its *K*_I_ values detailed in Table 2. Additionally, compounds **2f** with *para*‐bromo substituent and **2l** with *orto, para*‐dichloro substituent demonstrated significant inhibitory activity against BChE, with *K*_I_ values of 4.763 ± 0.321 μM and 5.738 ± 0.384 μM, respectively. Given its strong inhibition against both AChE and BChE, compound **2a** emerges as a promising selective inhibitor with potential therapeutic implications for AD treatment.

In the literature, several classes of compounds, including many triazole derivatives, have been synthesized and their inhibitory effects on cholinesterase enzymes have been extensively studied. These studies have contributed to the identification of therapeutically potent and selective inhibitors, providing important information for the development of effective cholinesterase inhibitors, especially for neurodegenerative diseases such as AD. In a study, new benzofuran thiazole derivatives were synthesized and the in vitro AChE and BChE inhibitory effects of these compounds were investigated. The compounds were reported to exhibit inhibitory potentials with IC_50_ values ranging from 4.42 ± 0.05 to 78.85 ± 0.69 µM for AChE and IC_50_ values ranging from 2.03 ± 0.62 to 81.28 ± 0.02 µM for BChE.^[^ ^38^ ^]^ In a study, a series of thiazolo and thiadiazolo [3,2‐a]pyrimidinone fused dihydrofuran compounds were synthesized and evaluated in vitro for their AChE inhibitory activities. The compounds were reported to exhibit inhibitory potential with IC_50_ values ranging from 0.15 ± 0.01 to >25 µM for AChE.^[^ ^39^ ^]^ The thiazole‐based furan derivatives synthesized in this study demonstrated enhanced inhibitory activity against cholinesterase enzymes compared to previously reported compounds. Notably, all other compounds except compound **2b** exhibited a higher degree of selectivity toward BChE rather than AChE, as evidenced by the selectivity index values presented in Table 1. These findings suggest that the newly synthesized derivatives possess promising potential as selective BChE inhibitors.

In general, molecular docking calculations are performed to support experimental studies and to determine the active sites of molecules. Molecular modeling is an important method used to study protein‐molecule interactions, and these interactions are analyzed by molecular docking calculations.^[^ ^40^ ^]^ This method is used to evaluate the activity of molecules against proteins and the interactions between them. The increase in the level of interaction indicates an increase in the biological activity of the molecules. As a result of the calculations, various parameters were obtained and each parameter provides information about different properties of the molecules.^[^ ^41^ ^]^ When these parameters are analyzed, the first parameter that determines the activity of the molecules is the docking score parameter.

When the in vitro and *in silico* findings of the compounds in Table 2 and **Figure** **1** are evaluated, it is understood that the compounds have a strong tendency to bind to active sites. Binding affinities and inhibitory activities of 13 newly synthesized compounds with AChE and BChE enzymes were evaluated. When docking scores and *K*_I_ values were examined, it was seen that there were significant differences between the binding capacities of the compounds to the enzymes. Since the docking score is negative, lower values indicate stronger binding. The lower the *K*_I_ value, the higher the binding power of the inhibitor with the enzyme.

Figure 1Protein–ligand interaction (2D and 3D). AChE and BChE (represented by 4EY7 and 4BDS) were subjected to molecular docking studies with compound **2a, 2b, 2f, 2l,** and tacrine.

**Figure:**
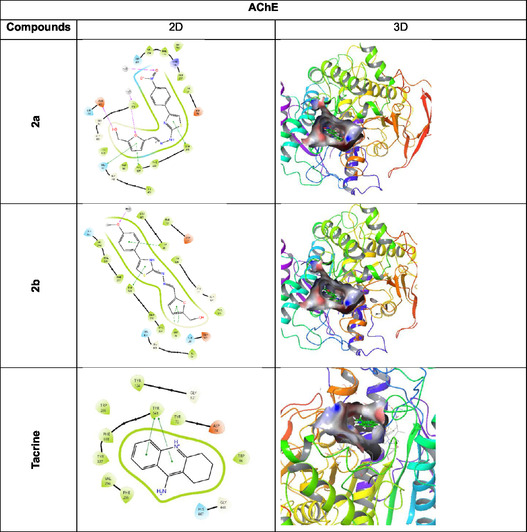

**Figure:**
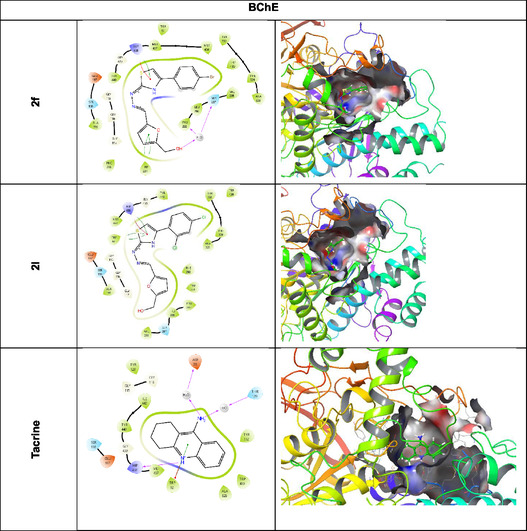

When the binding affinities with AChE enzyme were examined; compound **2i** (−9.181 kcal mol^−1^) and compound **2m** (−9.131 kcal mol^−1^) showed the highest binding affinity, while compound **2j** (−8.151 kcal mol^−1^) was calculated to have the lowest binding affinity. In general, the docking scores of the compounds are close to each other and vary between −8.1 kcal mol^−1^ and −9.2 kcal mol^−1^, that is, they all have a certain binding capacity. The compounds with the lowest *K*_I_ values are compound **2a** (14.887 µM) and compound **2b** (14.511 µM), which are the most potent inhibitors. The highest *K*_I_ value is compound **2m** (59.558 µM), which is one of the weakest inhibitors. Interestingly, the compound with the best docking score, compound **2i** (−9.181 kcal mol^−1^), shows a high *K*_I_ value (43.386 µM). This may suggest that although the compound has a good binding energy, its enzyme inhibition capacity may be low. In addition, the docking score indicates how strongly the compound binds to the active site of the enzyme. The *K*_I_ value indicates the enzyme inhibition capacity of the compound. Although compound **2i** has a low docking score (high binding affinity), its high *K*_I_ value may indicate that it binds tightly to the enzyme but its inhibitory activity at the active site is low. Compounds **2a** and **2b** are the strongest inhibitor candidates with both high docking scores and low *K*_I_ values (Table 1).

When the binding affinities with BChE enzyme were examined, the compounds showing the strongest binding were determined as compound **2j** (−7.837 kcal mol^−1^), compound **2l** (−7.732 kcal mol^−1^), compound **2d** (−7.637 kcal mol^−1^), compound **2i** (−7.596 kcal mol^−1^), compound **2h** (7.398 kcal mol^−1^), and compound **2k** (−7.294 kcal mol^−1^), respectively. In contrast, the compounds with the lowest binding affinities were determined as compound **2e** (−4.554 kcal mol^−1^) and compound **2c** (−5.377 kcal mol^−1^), respectively. In general, the docking scores of the compounds vary between −4.5 kcal mol^−1^ and −7.8 kcal mol^−1^, that is, there are significant differences between the binding capacities of the compounds. When the *K*_I_ values showing inhibitory activity were examined, it was found in the experimental results that the compounds with the lowest *K*_I_ values were compound **2f** (4.763 µM), compound **2l** (5.738 µM), compound **2a** (6.029 µM), compound **2i** (6.524 µM), and compound **2e** (6.436 µM), respectively. The compounds with the highest *K*_I_ values were compound **2b** (23.823 µM), compound **2m** (14.888 µM), compound **2j** (13.572 µM), and compound **2g** (12.875 µM), respectively. Compound **2j** (−7.837 kcal mol^−1^), although it has the best docking score (i.e., it binds well to the enzyme), has a very high *K*_I_ value (13.572 µM). This indicates that although the compound binds strongly to the enzyme, its inhibitory activity may be low. Compound **2f** has both a good docking score (−7.135 kcal mol^−1^) and the lowest *K*_I_ value (4.763 µM). This indicates that it is one of the strongest inhibitor candidates. Compound **2e** has a low docking score (−4.554), but a low *K*_I_ value (6.436 µM) (Table 1). This suggests that it may be effective as an inhibitor despite its weak binding to the enzyme. Compounds **2f** and **2l** may be the most effective inhibitors with both low docking scores and the lowest *K*_I_ values.

Inappropriate binding site, conformational changes or interactions with the enzyme surface that do not sufficiently support inhibitory activity may affect the inhibitory activity of compounds. Docking calculations generally do not take into account water molecules and are performed on a rigid structure. However, the flexibility of the enzyme in the real environment and its interactions with water molecules may affect the inhibitory activity of the compound. Therefore, even if compounds **2i** and **2j** bind strongly to the enzyme, they may be displaced by water molecules in the real biological environment or may not remain stable due to weak interactions. The compounds **2f** and **2l** are the most potent inhibitor candidates with low *K*_I_ values on BChE. The compound **2a** shows strong inhibitory activity against AChE and BChE. The compound **2m** is the compound with the lowest inhibitory activity against both enzymes. Compound **2i** has high binding affinity to AChE, but low inhibitory activity, but may be a better inhibitor against BChE with both strong binding and relatively low *K*_I_ value.

The compound **2a** interacts with the target protein 4EY7 by forming two hydrogen bonds (Glh202 and Tyr124) three *π*‐ bonds (Hıs447, Tyr337, and Tyr341) with the backbone residues, while the compound **2b** establishes one *π*‐ bond (Tyr341). The compound **2f** interacts with the target protein 4BDS by forming two hydrogen bonds (Pro285 and Ser287) with the backbone residues.

Crystal structure studies of the AChE (PDB ID: 4EY7 and 4BDS) have revealed the presence of distinct binding sites, including the aromatic patch (AP), oxyanionic hole, PAS, CAS, acyl site, and anionic site (AS). For the development of effective AChE inhibitors, inhibition of AChE activity at the AS and the PAS is crucial, as the accumulation of AChE‐bound amyloid peptide plaques in PAS contributes to pathological processes.^[^ ^42^, ^43^, ^44^, ^45^, ^46^, ^47^, ^48^, ^49^ ^–^ ^50^ ^]^ The CAS comprises the amino acid residues Trp86, Tyr133, Glu202, Ser203, Tyr337, Phe338, and His447, while the PAS includes Tyr72, Asp74, Tyr124, Trp286, Phe295, and Tyr341.^[^ ^51^, ^52^, ^53^ ^–^ ^54^ ^]^ Molecular docking analyses indicate that the PAS region interacts primarily with lipophilic groups through Trp286, whereas polar and basic groups bind to the CAS region via interactions with Trp86. Notably, the interaction with Trp286 is crucial for PAS binding, while interaction with Trp86 is essential for positioning within CAS. Consistent with molecular docking studies, compounds **2a** and **2b** demonstrated effective AChE inhibition by engaging in multiple binding interactions within both the PAS and CAS regions.

In the 2D interaction analysis of the compound **2a**, residues such as Tyr337, His447, and Trp86 are located within the CAS of the enzyme and directly interact with the ligand. Oxygen‐containing structures typically bind through hydrogen bonds and ionic interactions, highlighting the region where the enzyme's catalytic functions take place. Interactions between His447 and Tyr337 may facilitate the proper binding of the substrate to the active site. Additionally, residues Tyr72, Tyr124, and Asp74, which are part of the PAS, may support ligand binding. Amino acid residues in this region often stabilize ligand interactions through hydrogen bonding and *π*‐*π* interactions. The PAS region can regulate or inhibit substrate access to the active site.

In the 2D interaction analysis of the compound **2b**, the CAS is identified as the region where the enzyme binds its substrate and facilitates the reaction. Key catalytic residues, including His447, Tyr337, and Trp86, directly interact with the ligand. His447 plays a crucial role in proton transfer, further supporting the designation of this region as the CAS. Additionally, Tyr337 and Trp86 stabilize ligand binding through *π*‐*π* interactions. Regions where the ligand forms hydrogen bonds contribute to enzyme‐substrate interactions, suggesting that strong ligand binding to the CAS may influence the enzyme's reaction mechanism. If the ligand acts as an inhibitor, this binding may block enzymatic activity. The PAS typically regulates the access of substrates or inhibitors to the active site. Residues such as Tyr72, Tyr124, and Asp74 are located within the PAS, with Tyr124 and Asp74 being critical for ligand binding through hydrogen bonding or electrostatic interactions. If a ligand binds tightly to the PAS, it may prevent the enzyme's natural substrate from binding, thereby exerting an inhibitory effect.

In the interaction of the positive control tacrine molecule, Trp86 is an aromatic amino acid that typically interacts with ligands through *π*‐*π* stacking or hydrophobic interactions. In the given structure, Trp86 does not form a direct bond; however, it is likely to engage in *π*‐*π* stacking interactions with the aromatic rings of tacrine. This interaction plays a crucial role in stabilizing tacrine binding, thereby contributing to enzyme inhibition. Similarly, Phe295 is another aromatic amino acid capable of participating in hydrophobic interactions or *π*‐*π* stacking interactions. The cyclic structure of tacrine may allow it to fit into the hydrophobic region associated with Phe295, facilitating stable binding. These interactions help secure tacrine within the binding site, thereby enhancing its ability to inhibit ChE enzymes. The combined contributions of Trp86 and Phe295 to aromatic interactions, particularly *π*‐*π* stacking and hydrophobic interactions, play a key role in maintaining the ligand's position in the active site and increasing the effectiveness of enzyme inhibition.

The ligand interacts with both the CAS and PAS through various hydrogen bonds, *π*‐*π* interactions, and electrostatic interactions. Binding to the CAS can directly influence the enzyme's ability to process its substrate, while binding to the PAS may restrict access to the natural substrate, leading to indirect inhibition. If the ligand exhibits strong binding to the PAS, it may function as a noncompetitive inhibitor. It suggests that the compounds **2a** and **2b** can bind to both the active site and the peripheral site of the enzyme, thereby inhibiting its function.

ADME/T analysis (absorption, distribution, metabolism, excretion, and toxicity) was performed to examine the effects and responses of these studied molecules in human metabolism. With this analysis, the absorption of the molecules by human metabolism, their distribution in human metabolism, their excretion from metabolism, and finally, their toxicity values in metabolism were calculated. Many parameters that analyze the chemical properties of molecules are calculated, such as mol_MW (molar mass of molecules), molecular weight (MW), volume (molecular volume), LogP (The degree of lipophilicity of the molecule), topological polar surface area (TPSA; Refers to the polar surface area of the molecule, affects bioavailability), nRot (Number of rotationally free bonds)9, LogS (Degree of water solubility), and nHA and nHD ( Refers to the number of atoms that accept and give hydrogen bonds). The physicochemical and ADME properties of the synthesized compounds (compound **2a**, **2b**, **2f,** and **2l**) and tacrine are given in **Table** **3**.

**Table 3:** Physicochemical and ADME properties of synthesized compounds (**2a**, **2b**, **2f**, and **2l**) and tacrine.

	Compound 2a	Compound 2b	Compound 2f	Compound 2l	Tacrine	Optimal
Molecular weight (MW)	344.06	329.08	376.98	366.99	198.12	100–600
Volume	316.256	316.401	309.599	320.767	216.546	
Density	1.088	1.04	1.218	1.144	0.915	
nHA	8.0	6.0	5.0	5.0	2.0	0–12
nHD	2.0	2.0	2.0	2.0	2.0	0–7
nRot	6.0	6.0	5.0	5.0	0	0–11
nRing	3.0	3.0	3.0	3.0	3.0	0–6
MaxRing	6.0	6.0	6.0	6.0	14.0	0–18
nHet	9.0	7.0	7.0	8.0	2.0	1–15
fChar	0	0	0	0	0	−1
nRig	18.0	17.0	17.0	17.0	16.0	0–30
Flexibility	0.333	0.353	0.294	0.294	0	
Stereo centers	0	0	0	0	0	<2
TPSA	113.79	79.88	70.65	70.65	38.91	0–140
logS	−5.151	−4.808	−5.721	−5.968	−2.875	
logP	2.964	3.058	4.017	4.43	2.432	0–3
logD	3.149	3.25	3.748	3.881	2.101	11–3
Medicinal chemistry						
Lipinski rule	**	**	**	**	**	
Pfizer rule	**	**	*	*	**	
GSK rule	**	**	*	*	**	

* Rejected;

** Accepted.

The analyzed cytotoxicity data for compounds (**2a**, **2b**, **2f**, **2l,** and tacrine) show molecular weight of 344.06, 329.08, 376.98, 366.99, and 198.12 g/mol, TPSA of 113.79, 79.88, 70.65, 70.65, and 38.91 Å for both compounds, respectively. Orally active drugs transported transcellularly should not exceed a PSA of approximately 120 Å. A total polar surface ranging below 120 Å indicates good oral absorption and brain penetration.^[^ ^55^ ^,^ ^56^ ^]^ In the results, an appropriate number of rotatable bonds, H‐bond donors, H‐bond acceptors, and values indicating that most of the derivatives follow Lipinski's rule of 5 were found. Lipinski's rule of 5 is a quantitative approach for qualitative prediction of oral absorption. In terms of lipophilicity, most compounds displayed **logP** and **logD** values close to the optimal range (logP: 0–3), although compounds 2f and 2l exhibited slightly elevated values (logP > 4), which may indicate a tendency for lower aqueous solubility. This is supported by their **logS** values (e.g., –5.721 for 2f and –5.968 for 2l), suggesting limited solubility, a factor to consider in formulation development. Medicinal chemistry properties indicate that all four compounds passed **Lipinski**, **Pfizer**, and **GSK** rules, with compounds 2a and 2b showing full compliance (**), supporting their drug‐likeness potential.

TPSA is associated with hydrogen bonding of a molecule as a reliable predictor of bioavailability. Considering the drug‐like parameters predicted by ADME analysis that compounds (**2a**, **2b**, **2f**, **2l,** and tacrine) have TPSA in the optimum range of 113.79, 79.88, 70.65, 70.65, and 38.91 Å for compounds **2a, 2b, 2f, 2l,** and tacrine can be said to exhibit drug‐like behavior.

**Figure 2:**
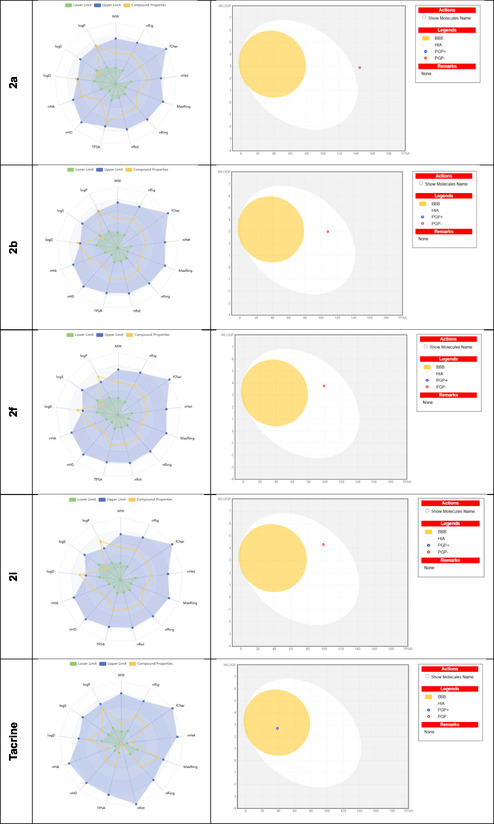
Boiled egg plot radar graph showing the chemical structure and physicochemical properties of compounds (**2a, 2b, 2f**, **2l**) and tacrine.

The chemical structure of (**2a, 2b, 2f** *,* **2l,** and tacrine) the physicochemical properties of this molecule are illustrated by a radar plot (spider plot). Molecular properties (e.g., LogP, LogS, nHD, TPSA) are expressed as a circle around it. Green line indicates the lower limit of the molecule's properties, yellow line indicates the compound properties, blue line indicates the lower limit of the molecule's properties. As seen in **Figure** **2**, the white region (albumin) of the boiled egg plot represents molecules with high gastrointestinal (GI) absorption potential, while the yellow region represents potential blood–brain barrier permeability. The gray area is reserved for molecules with low gastrointestinal absorption and low brain penetration. Compound **2a** found in the gray area has a low potential for GI absorption, suggesting that this compound is less likely to be bioavailable when administered orally.

## 3 Conclusion

Herein, 13 thiazole‐based furan derivatives (**2a‐2m**) were afforded and then characterized through ^1^H NMR and ^13^C NMR. Besides, all the compounds were screened for their cholinesterase inhibitory and antioxidant activities. Moreover, molecular docking studies were performed to elucidate the drug‐receptor interactions of the most potent compounds. Compounds **2b** and **2f** were identified as the most active inhibitors of AChE and BChE, respectively. Moreover, compounds **2a** and **2i** were found to be quite effective in terms of DPPH and ABTS values. *In silico* ADME/T profiling study was performed to explain the pharmacokinetic properties of the most effective compounds. In conclusion, this study will provide insight for researchers in their drug‐discovery studies for anti‐Alzheimer lead molecules.

In conclusion, the compounds synthesized in this study exhibited significant antioxidant potential as well as dual cholinesterase inhibitory activity. Notably, unlike traditional inhibitors such as donepezil and rivastigmine, our compounds also demonstrated measurable radical‐scavenging activity. Thus, synthesis compounds may provide additional neuroprotective benefits in AD by reducing oxidative stress, one of the causes of neurodegeneration. Furthermore, with their dual action (inhibition of ChE and antioxidant activity), these compounds may simultaneously address multiple pathological features of AD. Particularly, the high ACHE inhibition potential and antioxidant potential of compound 2a show that it is very promising in the treatment of AD. Although this study is limited to in vitro and *in silico* analyses, the promising activity of the synthesized compounds supports their potential as lead compounds for future in vivo evaluation.

## 4 Experimental Section

4.1

4.1.1

##### Chemistry: Synthesis of 2‐((5‐(hydroxymethyl)furan‐2‐Yl)methylene)hydrazine‐1‐Carbotioamide (1)

5‐(Hydroxymethyl)furan‐2‐carbaldehyde (5 g, 0.040) and thiosemicarbazide (3.61 g, 0.040 mol) were dissolved in ethanol (100 mL). The reaction mixture was heated under reflux conditions for three hours with continuous stirring, then chilled in an ice bath, and the precipitated product was filtered.

##### Chemistry: Synthesis of Target Compounds (2a‐2m)

Compound **1** (2.5 mmol) was treated with the appropriate 2‐bromoacetophenone derivative (2.5 mmol) in ethanol (50 mL). The reaction mixture was refluxed for about 5 h to afford furan‐based thiazole derivatives (**2a‐2m**). After completion of the reaction, the mixture was cooled in an ice bath, and the precipitated product was collected via filtration.

##### 4‐(4‐Nitrophenyl)‐2‐(2‐((5‐hydroxymethylfuran‐2‐yl)methylene)hydrazineyl)thiazole (2a)^[54]^

M.P.= 257.6 °C. ^1^H‐NMR (400 MHz, DMSO‐d_*6*_): *δ*: 4.46 (2H, s, CH_2_), 6.43 (1H, s, Aromatic CH), 6.77 (1H, s, Aromatic CH), 7.71 (1H, s, Aromatic CH), 7.91 (1H, s, Aromatic CH), 8.10 (2H, d, *J* = 7.80 Hz, 1,4‐disubstituted benzene), 8.28 (2H, d, *J* = 7.96 Hz, 1,4‐disubstituted benzene), 12.21 (1H, s, NH). ^13^C‐NMR (100 MHz, DMSO‐d_*6*_): *δ* = 56.48, 108.93, 109.74, 114.12, 124.58, 126.80, 132.70, 141.10, 146.69, 148.88, 148.91, 157.83, 168.71. HRMS (m/z): [M+H]^+^ calcd for C_15_H_12_N_4_O_4_S: 345.0652; found: 345.0666.

##### 4‐(4‐Methoxyphenyl)‐2‐(2‐((5‐Hydroxymethylfuran‐2‐Yl)methylene)hydrazineyl)thiazole (2b)

M.P. = >350 °C. ^1^H‐NMR (400 MHz, DMSO‐d_*6*_): *δ*: 3.73 (3H, s, OCH_3_), 4.33 (2H, s, CH_2_), 6.44 (1H, s, Aromatic CH), 6.77 (1H, s, Aromatic CH), 7.72 (1H, s, Aromatic CH), 7.90 (1H, s, Aromatic CH), 8.10 (2H, d, *J* = 7.20 Hz, 1,4‐disubstituted benzene), 8.28 (2H, d, *J* = 7.20 Hz, 1,4‐disubstituted benzene), 12.23 (1H, s, NH).^13^C‐NMR (100 MHz, DMSO‐d_*6*_): *δ* = 56.06, 56.61, 108.88, 109.99, 114.43, 124.96, 127.08, 132.81, 141.12, 146.47, 149.06, 157.83, 160.51, 168.55. HRMS (m/z): [M+H]^+^ calcd for C_16_H_12_N_4_O_2_S: 325.0754; found: 325.0763.

##### 4‐(4‐Cyanophenyl)‐2‐(2‐((5‐Hydroxymethylfuran‐2‐Yl)methylene)hydrazineyl)thiazole (2c)

M.P. = >350 °C. ^1^H‐NMR (400 MHz, DMSO‐d_*6*_): *δ*: 4.24 (2H, s, CH_2_), 6.20 (1H, s, Aromatic CH), 6.70 (1H, s, Aromatic CH), 7.21 (1H, s, Aromatic CH), 7.73–7.75 (2H, m, Aromatic CH), 7.76–7.78 (2H, m, Aromatic CH), 7.96 (1H, s, Aromatic CH).^13^C‐NMR (100 MHz, DMSO‐d_*6*_): *δ*= 56.48, 107.04, 110.92, 118.21, 121.35, 124.49, 127.36, 130.77, 132.93, 136.13, 148.23, 150.45, 156.08, 168.36.

##### 4‐(4‐Fluorophenyl)‐2‐(2‐((5‐Hydroxymethylfuran‐2‐Yl)methylene)hydrazineyl)thiazole (2d)

M.P. = >350 °C. ^1^H‐NMR (400 MHz, DMSO‐d_*6*_): *δ*: 4.40 (2H, s, CH_2_), 6.80 (1H, s, Aromatic CH), 7.16 (1H, s, Aromatic CH), 7.63–7.73 (2H, m, Aromatic CH), 8.14 (2H, s, Aromatic CH), 8.28–8.30 (1H, m, Aromatic CH), 8.64 (1H, s, Aromatic CH), 12.21 (1H, s, NH). ^13^C‐NMR (100 MHz, DMSO‐d_*6*_): *δ* = 56.48, 107.62, 111.47, 120.47, 121.66, 125.15, 131.77, 133.33, 135.90, 146.92, 150.04, 157.38, 169.78.

##### 4‐([1,1^′^‐Biphenyl]‐4‐Yl)‐2‐(2‐((5‐Hydroxymethylfuran‐2‐Yl)methylene)hydrazineyl) Thiazole (2e)

M.P. = >350 °C. ^1^H‐NMR (400 MHz, DMSO‐d_*6*_): *δ*: 4.96 (2H, s, CH_2_), 7.–417.56 (7H, m, Aromatic CH), 7.70–7.80 (6H, m, Aromatic CH). ^13^C‐NMR (100 MHz, DMSO‐d_*6*_): *δ* = 56.48, 110.87, 113.64, 125.89, 127.03, 127.57, 128.66, 129.45, 131.04, 132.13, 132.91, 134.01, 139.18, 140.38, 148.51, 150.54, 169.47.

##### 4‐(4‐Bromophenyl)‐2‐(2‐((5‐Hydroxymethylfuran‐2‐Yl)methylene)hydrazineyl)thiazole (2f)

M.P. = >350 °C. ^1^H‐NMR (400 MHz, DMSO‐d_*6*_): *δ*: 4.42 (2H, s, CH_2_), 7.19–7.52 (6H, m, Aromatic CH), 7.90–8.12 (2H, m, Aromatic CH). ^13^C‐NMR (100 MHz, DMSO‐d_*6*_): *δ* = 56.48, 107.62, 111.47, 120.47, 121.66, 125.15, 131.77, 133.33, 135.90, 146.92, 150.04, 157.38, 169.78.

##### 4‐(4‐Methylphenyl)‐2‐(2‐((5‐Hydroxymethylfuran‐2‐Yl)methylene)hydrazineyl)thiazole (2g)

M.P. = >350 °C. ^1^H‐NMR (400 MHz, DMSO‐d_6_): *δ*: 2.33 (3H, s, CH_3_), 4.41 (2H, s, CH_2_), 7.12–7.25 (5H, m, Aromatic CH), 7.43–7.85 (3H, m, Aromatic CH).^13^C‐NMR (100 MHz, DMSO‐d_*6*_): *δ* = 21.24, 56.34, 116.92, 119.60, 122.74, 125.23, 127.26, 129.67, 131.14, 135.02, 148.32, 151.65, 157.19, 168.55. HRMS (m/z): [M+H]^+^ calcd for C_16_H_15_N_3_O_2_S: 314.0958; found: 314.0966.

##### 4‐(3,4‐Dichlorophenyl)‐2‐(2‐((5‐Hydroxymethylfuran‐2‐Yl)methylene)hydrazineyl) Thiazole (2H)

M.P. = >350 °C. ^1^H‐NMR (400 MHz, DMSO‐d_*6*_): *δ*: 4.22 (2H, s, CH_2_), 6.30–6.34 (1H, m, Aromatic CH), 6.68 (1H, s, Aromatic CH), 7.62–7.81 (5H, m, Aromatic CH), 12.11 (1H, s, NH). ^13^C‐NMR (100 MHz, DMSO‐d_*6*_): *δ* = 56.48, 108.33, 110.83, 117.01, 120.52, 123.11, 127.36, 131.14, 131.68, 134.65, 135.76, 137.79, 150.91, 156.36, 169.84.

##### 4‐(2,4‐Difluorophenyl)‐2‐(2‐((5‐Hydroxymethylfuran‐2‐Yl)methylene)hydrazineyl) Thiazole (2i)

M.P. = >350 °C. ^1^H‐NMR (400 MHz, DMSO‐d_*6*_): *δ*: 4.37 (2H, s, CH_2_), 6.29 (1H, s, Aromatic CH), 6.70 (1H, s, Aromatic CH), 7.16–7.35 (3H, m, Aromatic CH), 7.60 (1H, br.s, Aromatic CH), 7.81–7.84 (1H, m, Aromatic CH). ^13^C‐NMR (100 MHz, DMSO‐d_*6*_): *δ* = 56.47, 108.52, 112.12, 116.92, 122.37, 124.31, 126.06, 130.03, 134.65, 145.46, 148.51, 155.89, 159.13, 162.45, 169.10.

##### 4‐Phenyl‐2‐(2‐((5‐hydroxymethylfuran‐2‐yl)methylene)hydrazineyl)thiazole (2j)^[^ ^57^ ^]^

M.P. = >350 °C. ^1^H‐NMR (400 MHz, DMSO‐d_6_): *δ*: 4.42 (2H, s, CH_2_), 6.73 (1H, s, Aromatic CH), 7.25–7.42 (5H, m, Aromatic CH), 7.64–7.65 (1H, m, Aromatic CH), 7.86 (1H, br.s, Aromatic CH).^13^C‐NMR (100 MHz, DMSO‐d_6_): *δ* = 56.89, 113.13, 120.06, 124.22, 128.19, 128.98, 131.33, 134.56, 141.58, 147.67, 150.54, 156.91, 168.36. HRMS (m/z): [M+H]^+^ calcd for C_15_H_13_N_3_O_2_S: 300.0801; found: 300.0805.

##### 4‐(4‐Chlorophenyl)‐2‐(2‐((5‐hydroxymethylfuran‐2‐yl)methylene)hydrazineyl)thiazole (2k)^[^ ^57^ ^]^

M.P. = >350 °C. ^1^H‐NMR (400 MHz, DMSO‐d_*6*_): *δ*: 4.37 (2H, s, CH_2_), 6.78–6.79 (1H, m, Aromatic CH), 7.15–7.17 (1H, m, Aromatic CH), 7.61 (1H, s, Aromatic CH), 7.84–7.88 (2H, m, Aromatic CH), 8.00–8.03 (3H, m, Aromatic CH).^13^C‐NMR (100 MHz, DMSO‐d_*6*_): *δ* = 56.48, 107.50, 111.47, 120.98, 124.59, 128.93, 130.08, 131.86, 134.93, 147.31, 151.37, 156.26, 168.55.

##### 4‐(2,4‐Dichlorophenyl)‐2‐(2‐((5‐Hydroxymethylfuran‐2‐Yl)methylene)hydrazineyl) Thiazole (2l)

M.P. = 212.4 °C. ^1^H‐NMR (400 MHz, DMSO‐d_*6*_): *δ*: 4.43 (2H, s, CH_2_), 7.50–7.51 (3H, m, Aromatic CH), 7.70 (2H, s, Aromatic CH), 7.82–7.88 (2H, m, Aromatic CH), 12.11 (1H, s, NH).^13^C‐NMR (100 MHz, DMSOd_*6*_): *δ* = 56.15, 110.36, 120.33, 122.60, 124.34, 126.77, 128.03, 129.94, 130.18, 132.17, 134.70, 148.02, 150.44, 155.36, 168.04.

##### 4‐(3‐Nitrophenyl)‐2‐(2‐((5‐Hydroxymethylfuran‐2‐Yl)methylene)hydrazineyl)thiazole (2m)

M.P. = 182.6 °C. ^1^H‐NMR (400 MHz, DMSO‐d_*6*_): *δ*: 4.42 (2H, s, CH_2_), 6.39–6.51 (1H, m, Aromatic CH), 6.77–6.78 (2H, m, Aromatic CH), 7.71–7.86 (2H, m, Aromatic CH), 8.14–8.21 (2H, m, Aromatic CH), 8.66 (1H, s, Aromatic CH), 12.23 (1H, s, NH). ^13^C‐NMR (100 MHz, DMSO‐d_*6*_): *δ* = 56.89, 111.47, 115.07, 120.71, 122.92, 126.53, 128.65, 132.25, 134.47, 136.78, 145.18, 148.32, 150.63, 156.82, 168.08.

##### Biological Activity: Radical Scavenging Activity

The methods outlined by Blois and Re were used to assess the DPPH^**·**^ and ABTS^+·^ free radical scavenging activities, respectively.^[^ ^58^, ^59^ ^–^ ^60^ ^]^ A series of stock concentrations of synthesized thiazole‐based furan derivatives (**2a‐2m**) was prepared. For DPPH assay, a radical solution of 1 mM DPPH solution was prepared. Final concentrations were adjusted by diluting the sample stock solutions with ethanol to a total volume of 2 mL and then adding 0.5 mL of DPPH solution to each tube. The mixtures were incubated at room temperature and protected from light for 30 min. Absorbance was measured at 517 nm. In the ABTS test, 7 mM ABTS solution was prepared, and radical cations were formed by adding 2.45 mM persulfate. The absorbance of the control solution was adjusted to 0.700 ± 0.025 at 734 nm. For analysis, 0.5 mL of ABTS^+·^ radical solution was added to each sample. The absorbance at 734 nm was then measured against the blank solution following a 30 min incubation period.

##### Biological Activity: Cholinesterase Activity

A modified version of the Ellman et al. (1961) method was applied to measure ChE activity.^[^ ^61^ ^]^ Activity variations were tracked at 37 °C using acetylthiocholine iodide (ATChI) as a substrate for AChE (from electric eels) and butyrylcholine iodide (BChI) as a substrate for BChE (from horse serum). The enzyme solution (5.32 × 10^−3^ U, 50 µL) was added after the test was carried out in a cuvette that contained Tris–HCl buffer (100 µL, 1 m; pH = 8.0), DTNB solution (0.01 m, 100 µL), and ATChI/BChI solutions (0.050 m, 50 µL). A spectrophotometer was used to detect absorbance at 412 nm following a 5‐minute incubation period; each assay was carried out in triplicate.^[^ ^62^ ^,^ ^63^ ^]^

##### Biological Activity: in Vitro Inhibition Studies

The inhibitor compounds’ stock solutions were made in 20% DMSO. The thiazole‐based furan derivatives were tested at a minimum of five distinct inhibitor concentrations, usually between 10^–2^ and 10^2^ μM, to determine cholinesterase activity inhibition ranging from 0% to 100%. Using at least five different inhibitor concentrations against the AChE and BChE enzymes, the inhibitory effects of thiazole‐based furan derivatives were assessed across a range of inhibitor doses. The activity % versus inhibitor concentration graphs were used to determine the derivatives’ IC_50_ values. Furthermore, Lineweaver–Burk plots were used to assess inhibition types and inhibition constants (*K*_I_), providing thorough information on the kinetic behavior of these inhibitors.^[^ ^64^ ^,^ ^65^ ^]^

##### Biological Activity: Molecular Docking Calculation

The molecular docking method was used to evaluate the activity of molecules toward biological materials. Crystal structures of AChE (PDB ID: 4EY7, resolution: 2.35 Å) and BChE (PDB ID: 4BDS, resolution: 2.10 Å) were taken from the PDB database. Molecular docking calculations were performed with Schrödinger's Maestro software.^[^ ^66^ ^]^ The relevant module was used for protein preparation and the LigPrep module was used for ligand preparation. Prepared proteins and molecules were processed for interaction analysis by the Glide ligand docking method.^[^ ^67^ ^]^

##### Biological Activity: ADME Analysis

The prediction of ADME of the most bioactive of the synthesized thiazole‐based furan derivatives (**2a, 2b, 2f and 2l**) was performed using the online tools of Swiss ADME (http://www.swissadme.ch/) and Admetlab (https://admetmesh.scbdd.com/). The canonical SMILES format of these compounds was created with ChemDraw. Within the scope of the analysis, physicochemical properties such as lipophilicity, drug similarity, pharmacokinetic properties, TPSA, number of rotatable bonds and compliance with Lipinski's rule of five were evaluated. In addition, ADME/T analysis was performed to determine the potential effects of the molecules investigated on human metabolism.

##### Statistical Analysis

Statistical analyses and graphical visualizations were conducted using GraphPad Prism version 8.0 (GraphPad Software Inc., La Jolla, CA, USA), a widely adopted software in biological and pharmaceutical research due to its robust analytical capabilities. Enzyme inhibition and antioxidant experiments were performed in three independent replicates. The data were evaluated using descriptive statistical methods, and the results are expressed as mean ± standard error of the mean, providing an estimate of the variability around the average values.

## Conflict of Interest

The authors declare no conflict of interest.

## Supporting information

Supplementary Material

## Data Availability

The data that support the findings of this study are available from the corresponding author upon reasonable request.
