# Supplementary material for: Design, Synthesis, In Silico Absorption, Distribution, Metabolism, and Elimination and Molecular Docking Studies of Thiazole‐Based Furan Derivatives, and Their Biological Evaluation for Alzheimer Disease Therapy
Source: ChemistryOpen. 2025 Aug 10;14(12):e202500305. doi: 10.1002/open.202500305 (PMC12680558; doi:10.1002/open.202500305)
Supplement: Supplementary file 1 — Supplementary Material [file OPEN-14-e202500305-s001.pdf]

Design, Synthesis, In silico ADME and Molecular Docking Studies of Thiazole-Based Furan Derivatives, and Their Biological Evaluation for Alzheimer's Disease Therapy

Abdüllatif Karakaya,[a, b] Ulviye Acar Çevik, [c] Betül Kaya, [d] Bilge Çiftçi, [e] Adem Necip, [f] Mesut Işık,*[g] Şükrü Beydemir, *[h] Yusuf Özkay, [c] Zafer Asım Kaplancıklı, [c, ı]

[a] Department of Pharmaceutical Chemistry, Faculty of Pharmacy, Zonguldak Bulent Ecevit University, 67600, Zonguldak, Turkey

[b] Institute of Graduate Education, Anadolu University, 26470, Eskişehir, Turkey

[c] Department of Pharmaceutical Chemistry, Faculty of Pharmacy, Anadolu University, 26470, Eskişehir, Turkey

[d] Vocational School of Health Services, Pharmacy Services, Bilecik Şeyh Edebali University, 11230, Bilecik, Turkey

[e] Vocational School of Health Services, Bilecik Şeyh Edebali University, 11230, Bilecik, Turkey

[f] Department of Pharmacy Services, Vocational School of Health Services, Harran University, 63300, Şanlıurfa, Turkey

[g] Department of Bioengineering, Faculty of Engineering, Bilecik Şeyh Edebali University, 11230, Bilecik, Turkey

E-mail: mesut.isik@bilecik.edu.tr

[h] Department of Biochemistry, Faculty of Pharmacy, Anadolu University, 26470, Eskişehir, Turkey

E-mail: sukrubeydemir@anadolu.edu.tr

[ı] The Rectorate of Bilecik Şeyh Edebali University, 11230, Bilecik, Turkey

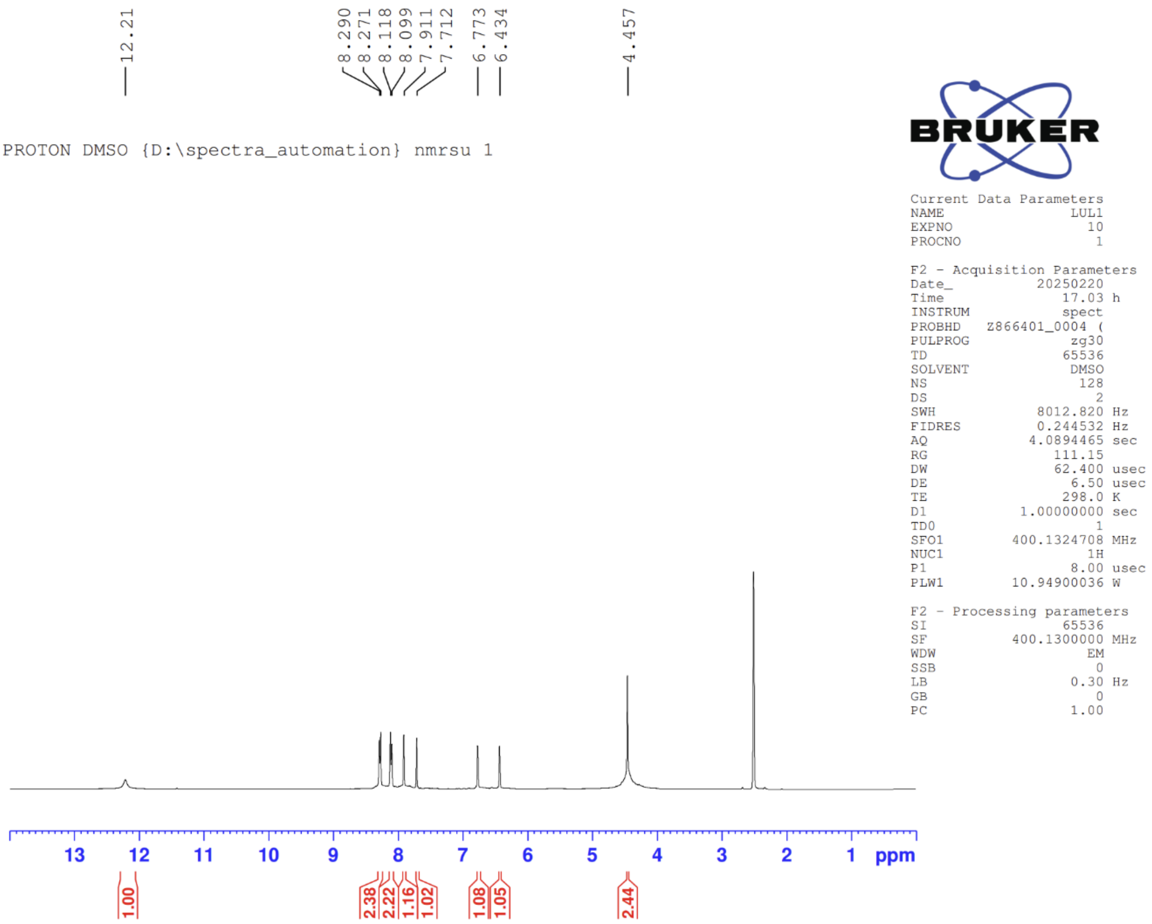

Figure S1. ^{1}H-NMR spectrum of compound **2a**

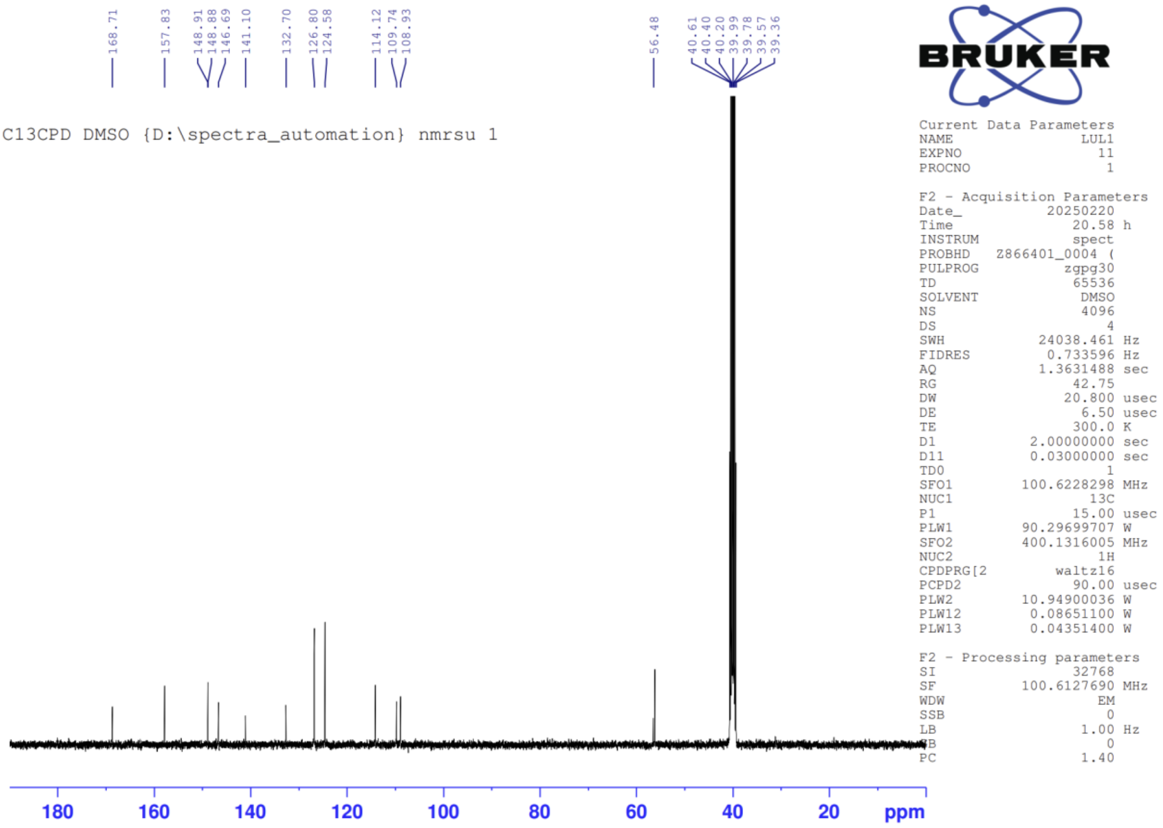

Figure S2. ^{13}C-NMR spectrum of compound **2a**

Data File: C:\LabSolutions\Data\Analiz\uac\LUL-1_137.lcd

Elmt	Val.	Min	Max	Elmt	Val.	Min	Max	Elmt	Val.	Min	Max	Elmt	Val.	Min	Max	Use Adduct
H	1	5	35	O	2	0	5	S	2	0	1	Ru	2	0	0	H
C	4	5	26	F	1	0	0	Cl	1	0	0	Pd	2	0	0	
N	3	0	5	P	3	0	0	Br	1	0	0	I	3	0	0	

Error Margin (ppm):	5	DBE Range:	10.0 - 20.0	Electron Ions:	both
HC Ratio:	unlimited	Apply N Rule:	yes	Use MSn Info:	yes
Max Isotopes:	3	Isotope RI (%):	1.00	Isotope Res:	9000
MSn Iso RI (%):	10.00	MSn Logic Mode:	AND	Max Results:	500

Event#: 1 MS(E+) Ret. Time : 2.947 -> 3.267 Scan# : 443 -> 491

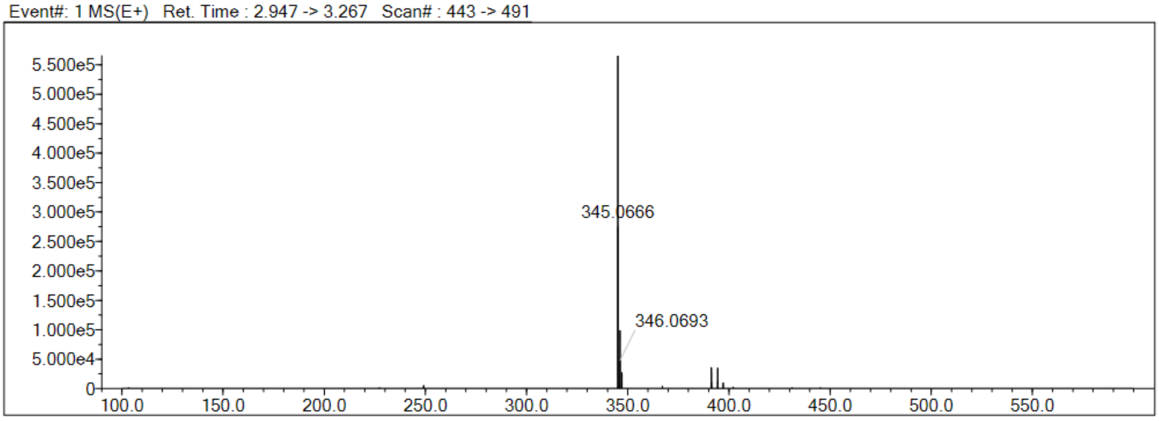

Measured region for 345.0666 m/z

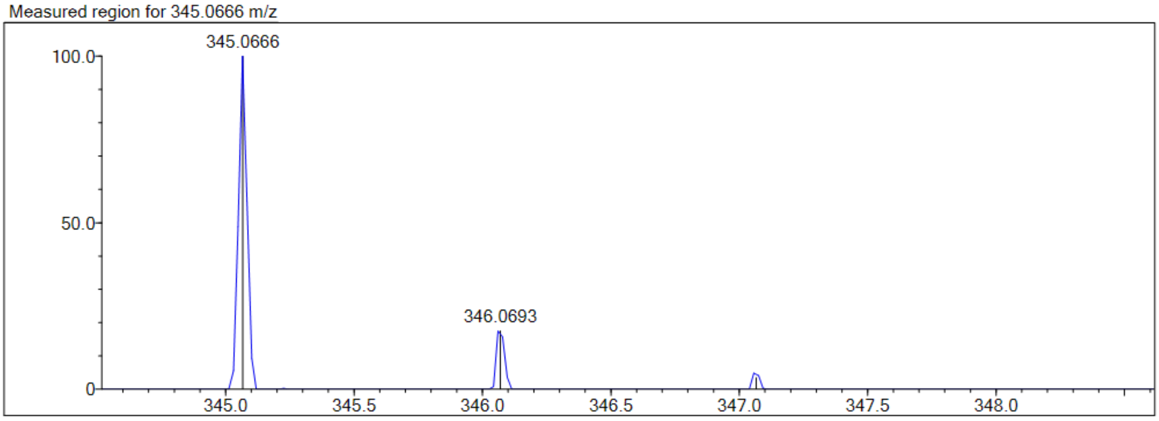

C15 H12 N4 O4 S [M+H]+ : Predicted region for 345.0652 m/z

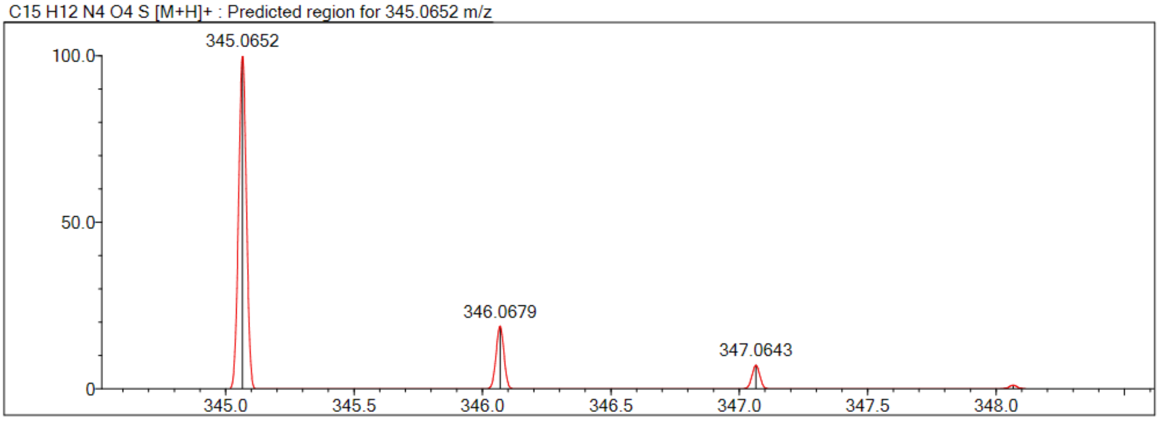

Rank	Score	Formula (M)	Ion	Meas. m/z	Pred. m/z	Df. (mDa)	Df. (ppm)	Iso	DBE
1	82.82	C15 H12 N4 O4 S	[M+H]+	345.0666	345.0652	1.4	4.06	89.68	12.0

Figure S3. Mass spectrum of compound **2a**

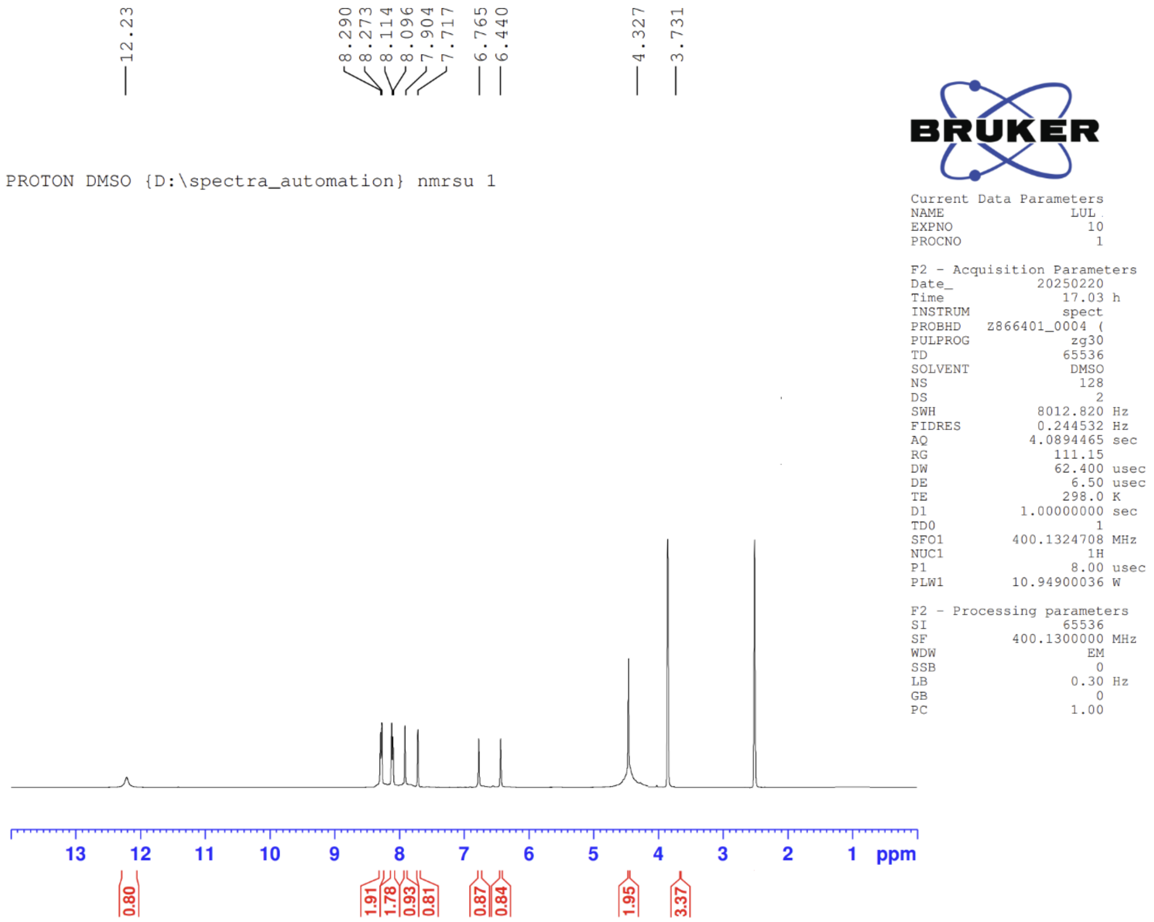

Figure S4. ^{1}H-NMR spectrum of compound **2b**

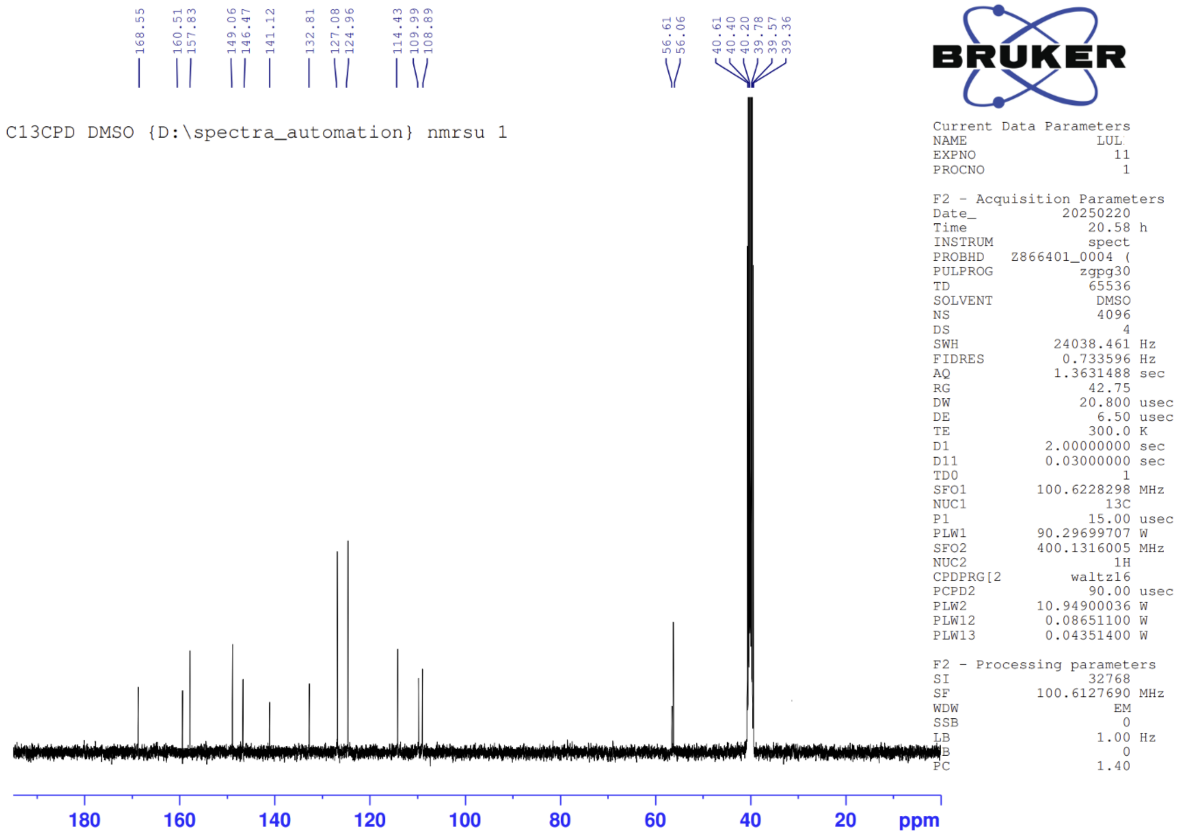

Figure S5. ^{13}C-NMR spectrum of compound **2b**

Formula Predictor Report - LUL-3_139.lcd

Page 1 of 1

Data File: C:\LabSolutions\Data\Analiz\uac\LUL-3_139.lcd

Elmt	Val.	Min	Max	Elmt	Val.	Min	Max	Elmt	Val.	Min	Max	Elmt	Val.	Min	Max	Use Adduct
H	1	5	35	O	2	0	5	S	2	0	1	Ru	2	0	0	H
C	4	5	26	F	1	0	0	Cl	1	0	0	Pd	2	0	0	
N	3	0	5	P	3	0	0	Br	1	0	0	I	3	0	0	

Error Margin (ppm):	5	DBE Range:	10.0 - 20.0	Electron Ions:	both
HC Ratio:	unlimited	Apply N Rule:	yes	Use MSn Info:	yes
Max Isotopes:	3	Isotope RI (%):	1.00	Isotope Res:	9000
MSn Iso RI (%):	10.00	MSn Logic Mode:	AND	Max Results:	500

Event#: 1 MS(E+) Ret. Time : 2.080 -> 2.400 Scan# : 313 -> 361

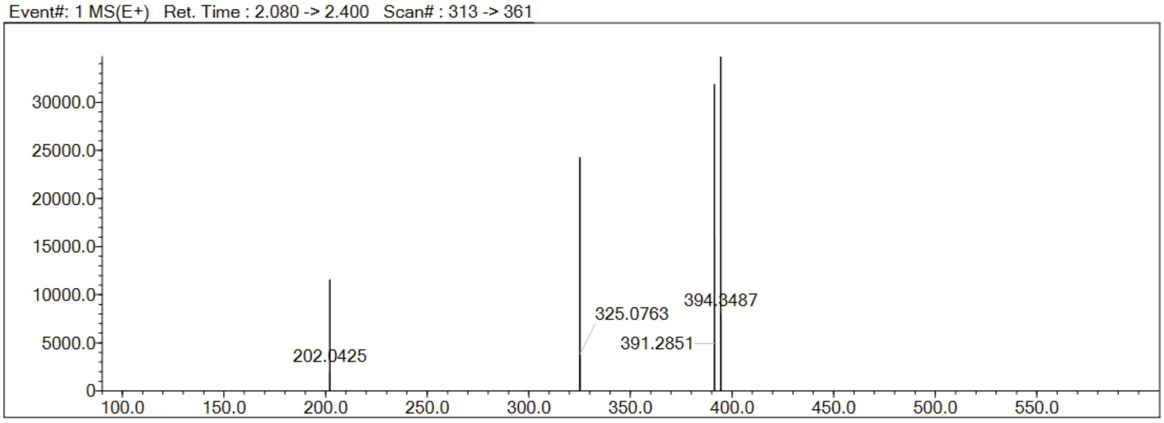

Measured region for 325.0763 m/z

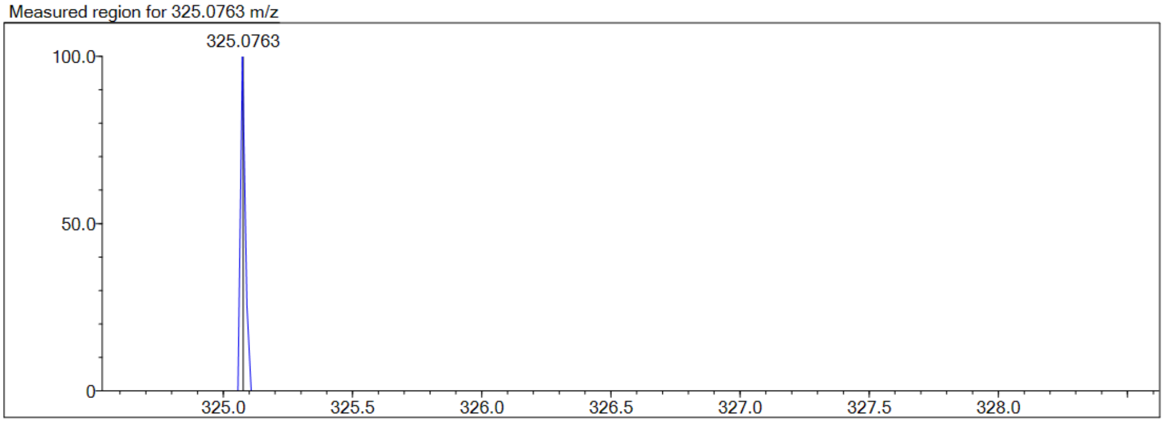

C16 H12 N4 O2 S [M+H]+ : Predicted region for 325.0754 m/z

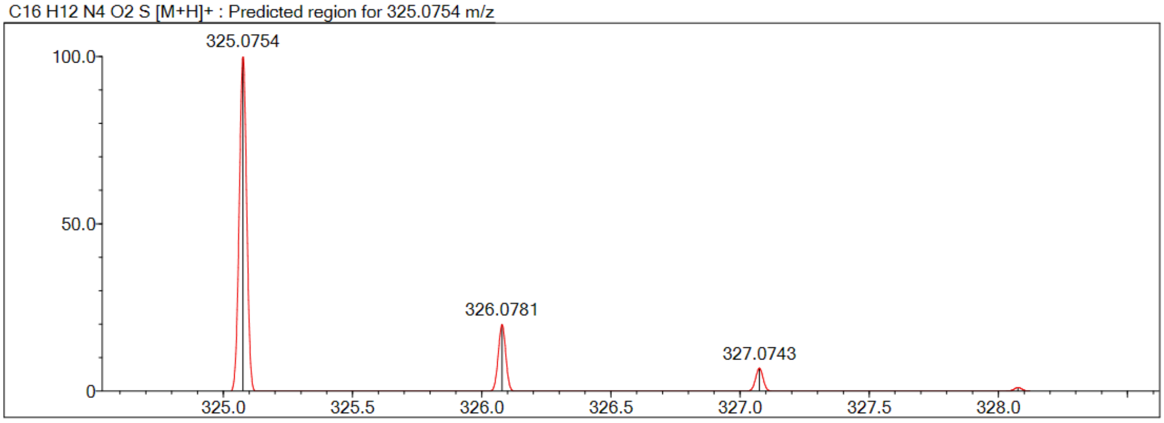

Rank	Score	Formula (M)	Ion	Meas. m/z	Pred. m/z	Df. (mDa)	Df. (ppm)	Iso	DBE
1	0.00	C16 H12 N4 O2 S	[M+H]+	325.0763	325.0754	0.9	2.77	0.00	13.0

Figure S6. Mass spectrum of compound **2b**

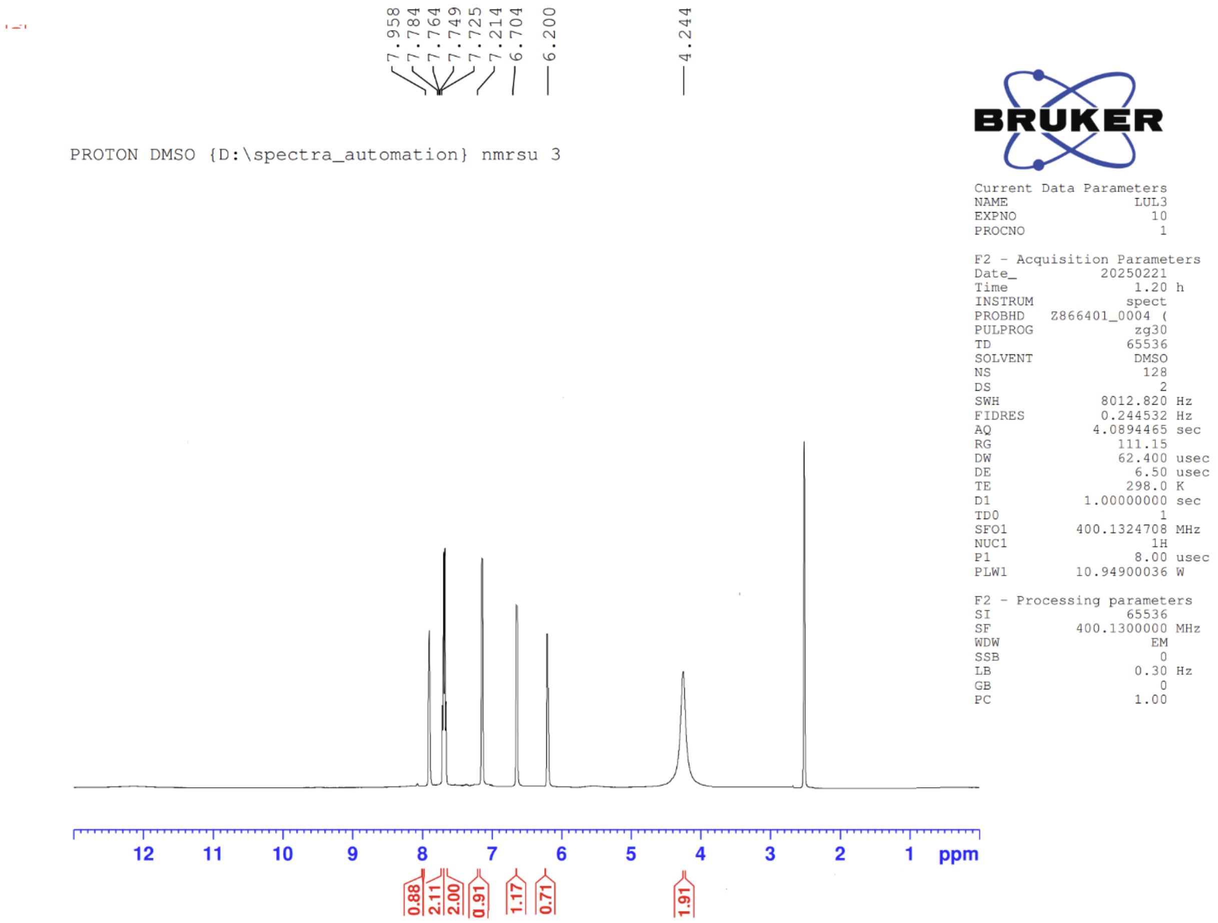

Figure S7. ^{1}H-NMR spectrum of compound **2c**

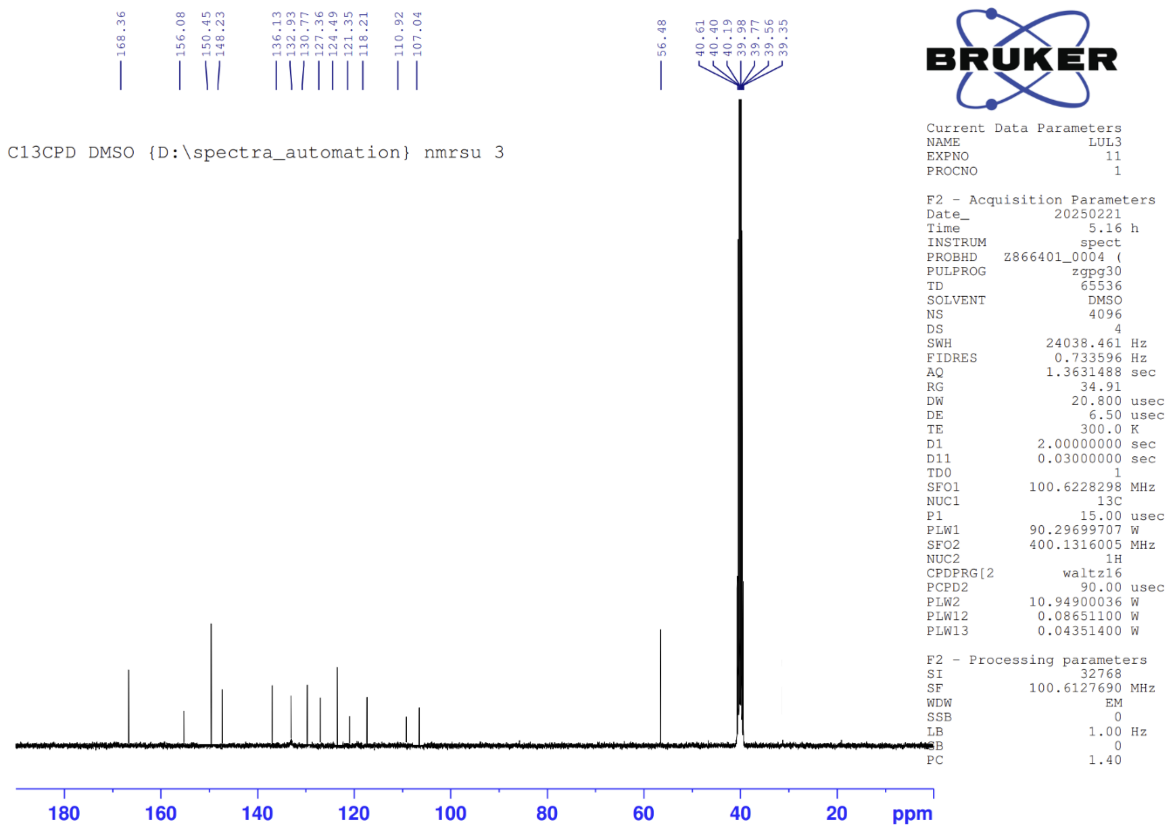

Figure S8. ^{13}C-NMR spectrum of compound **2c**

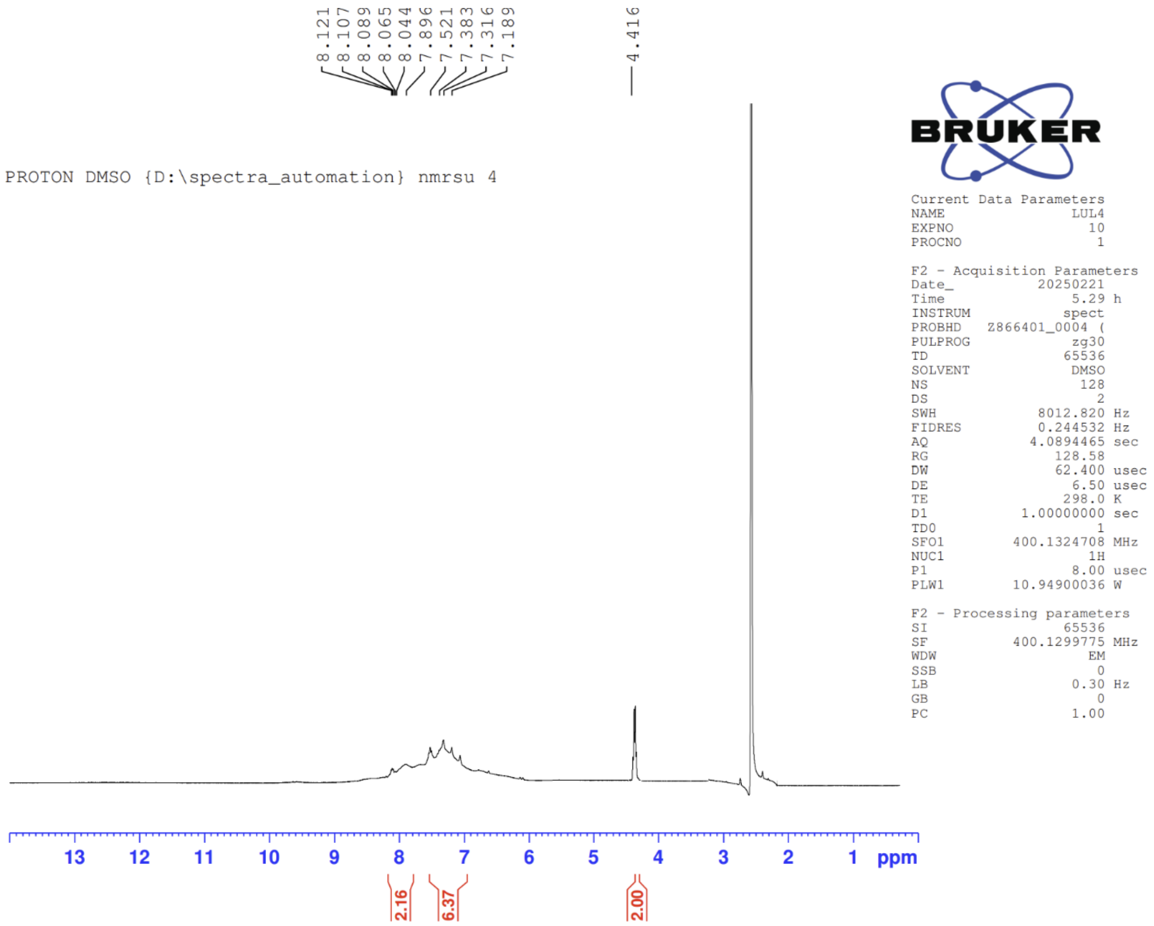

Figure S9. ^{1}H-NMR spectrum of compound **2d**

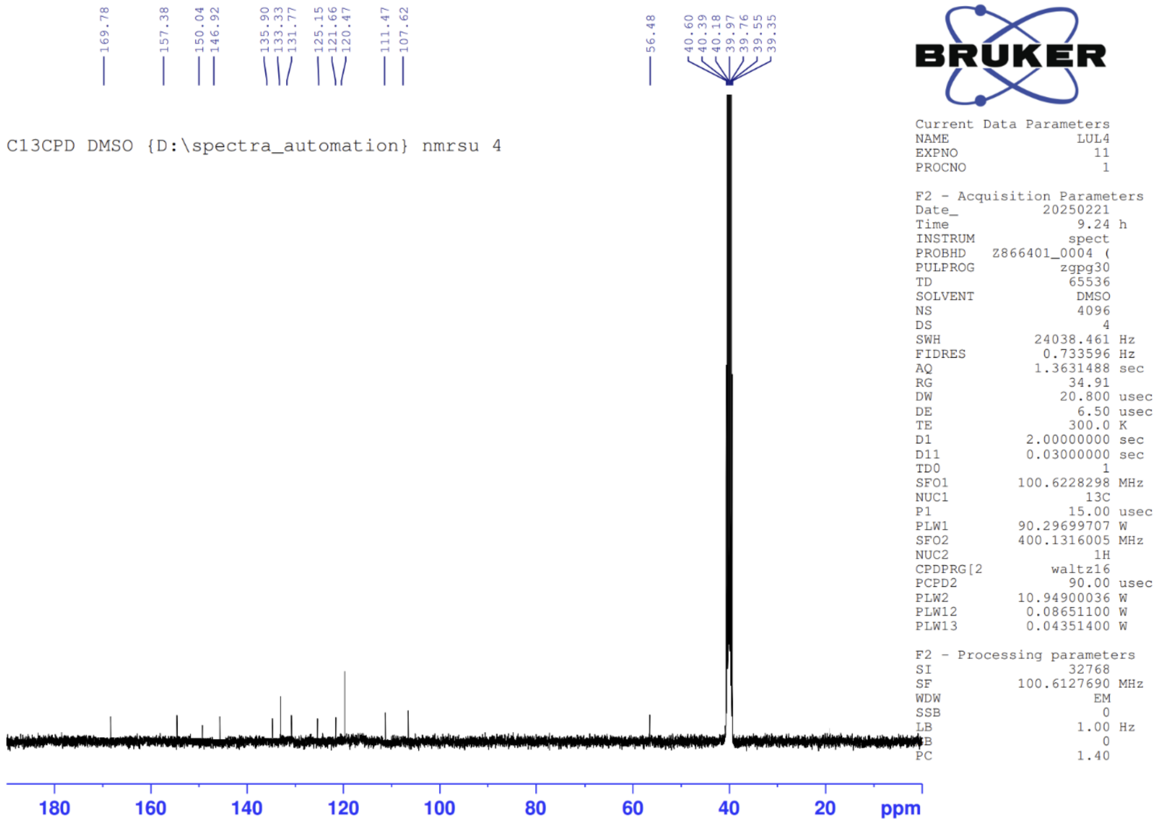

Figure S10. ^{13}C-NMR spectrum of compound **2d**

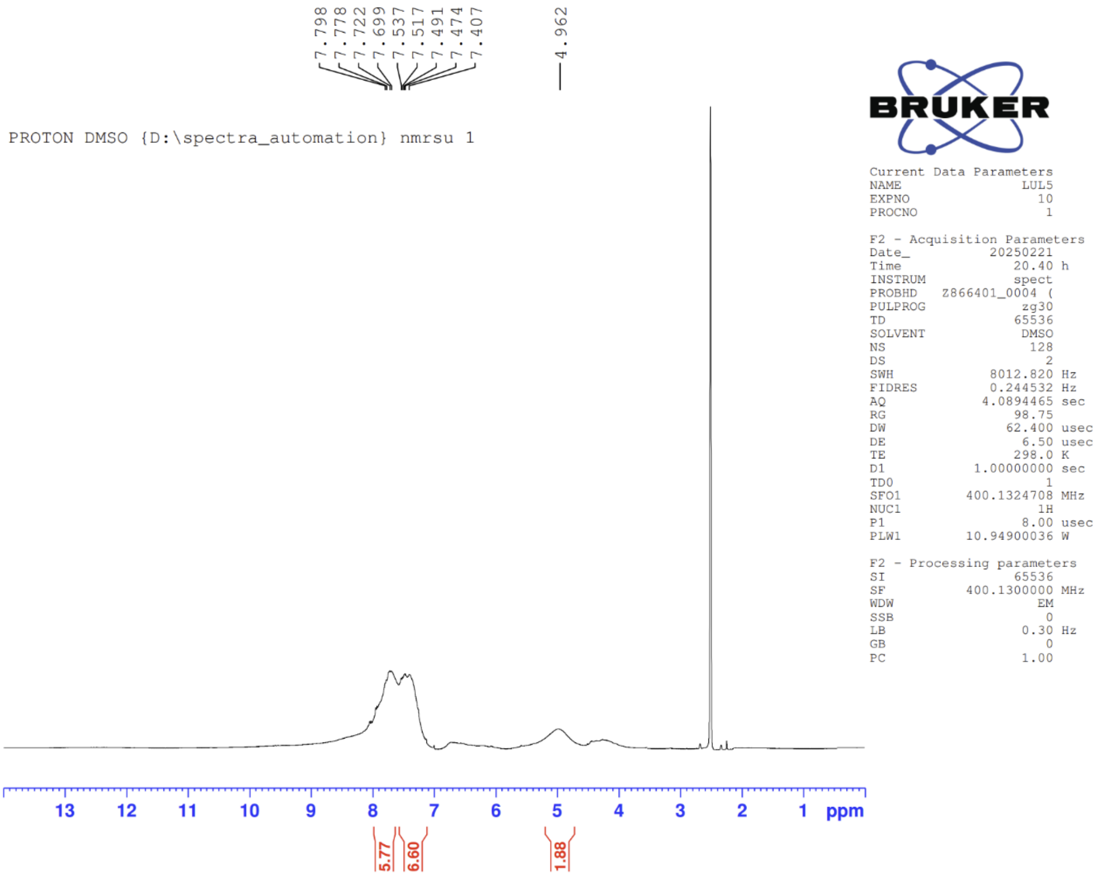

Figure S11. ^{1}H-NMR spectrum of compound **2e**

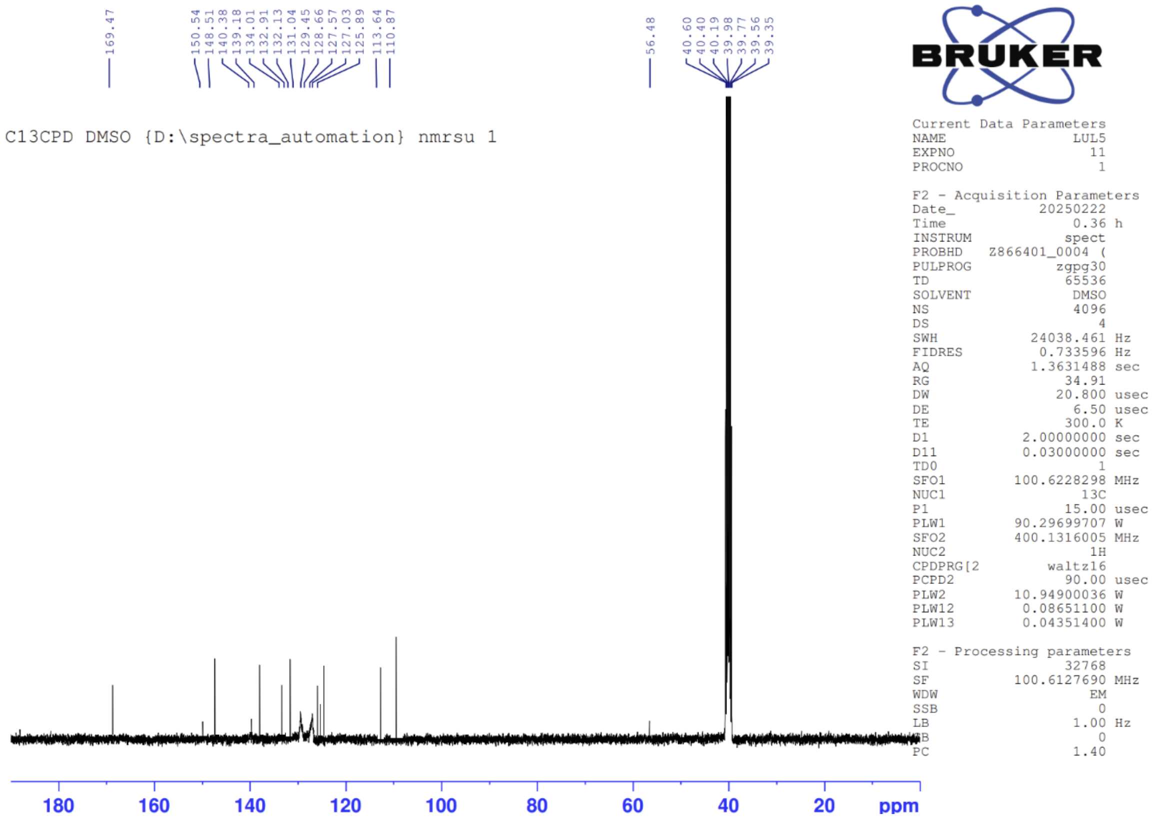

Figure S12. ^{13}C-NMR spectrum of compound **2e**

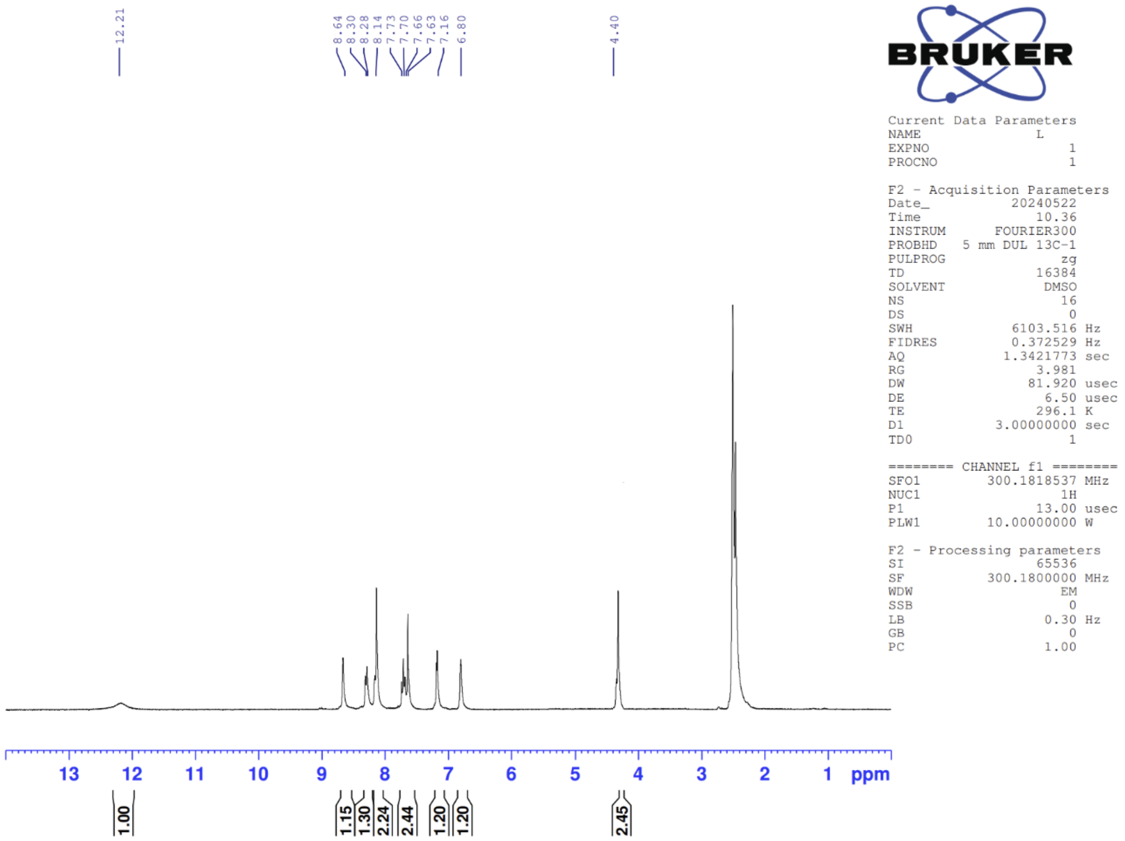

Figure S13. ^{1}H-NMR spectrum of compound **2f**

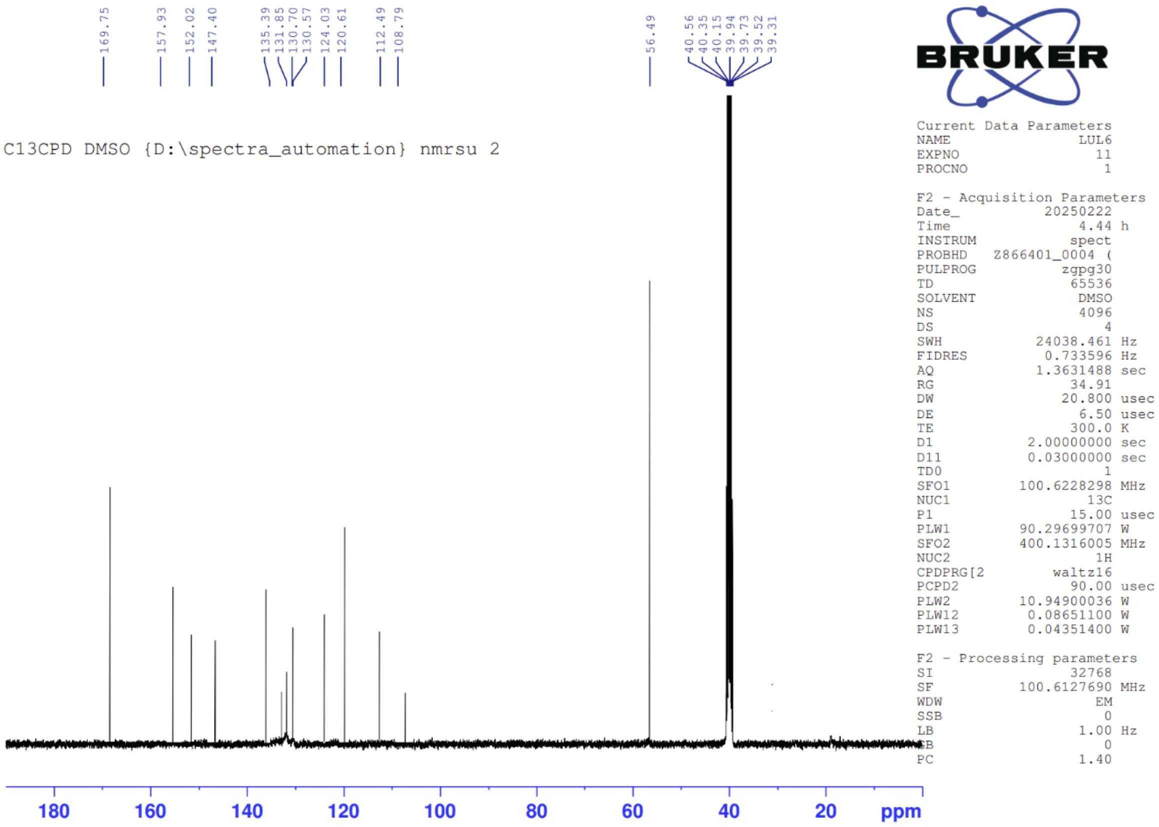

Figure S14. ^{13}C-NMR spectrum of compound **2f**

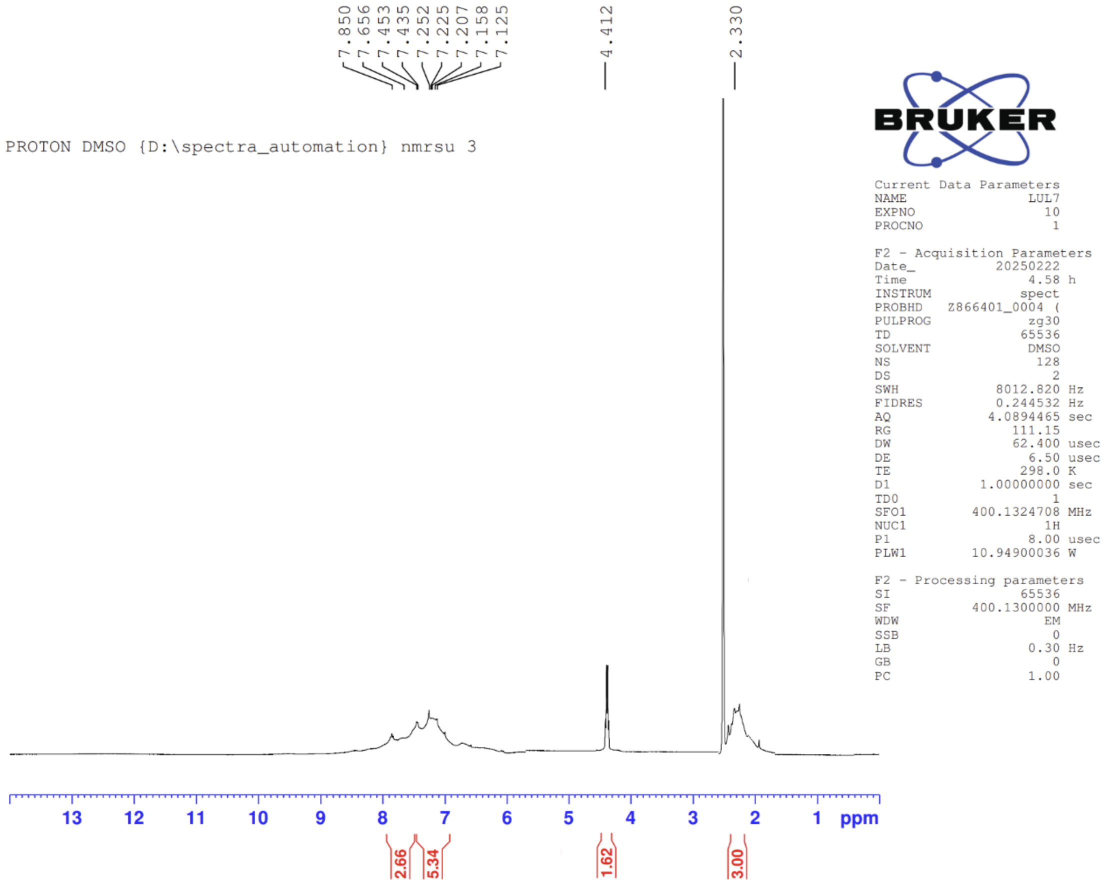

Figure S15. ^{1}H-NMR spectrum of compound **2g**

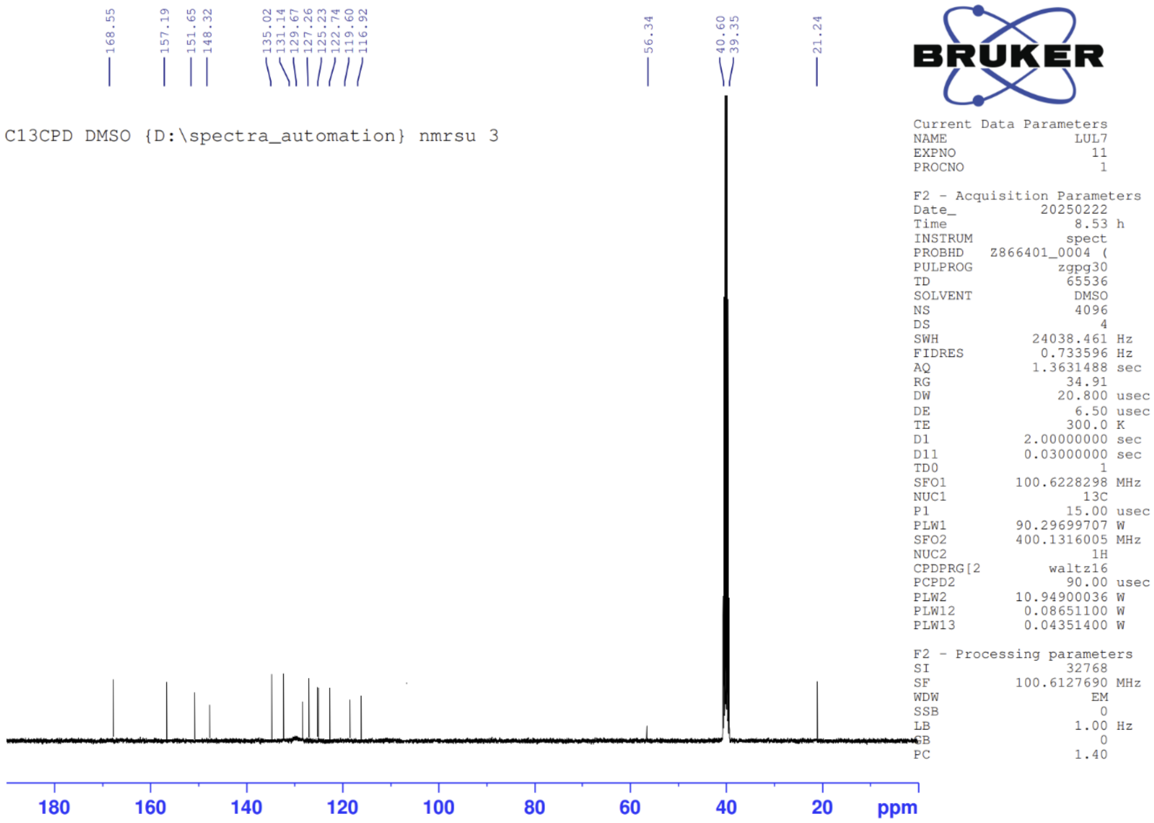

Figure S16. ^{13}C-NMR spectrum of compound **2g**

Data File: C:\LabSolutions\Data\Analiz\uac\LUL-7_143.lcd

Elmt	Val.	Min	Max	Elmt	Val.	Min	Max	Elmt	Val.	Min	Max	Elmt	Val.	Min	Max	Use Adduct
H	1	5	35	O	2	0	5	S	2	1	1	Ru	2	0	0	H
C	4	5	26	F	1	0	0	Cl	1	0	0	Pd	2	0	0	
N	3	0	5	P	3	0	0	Br	1	0	0	I	3	0	0	

Error Margin (ppm): 5
HC Ratio: unlimited
Max Isotopes: 3
MSn Iso RI (%): 10.00

DBE Range: 10.0 - 20.0
Apply N Rule: yes
Isotope RI (%): 1.00
MSn Logic Mode: AND

Electron Ions: both
Use MSn Info: yes
Isotope Res: 9000
Max Results: 500

Event#: 1 MS(E+) Ret. Time : 3.480 -> 3.760 Scan# : 523 -> 565

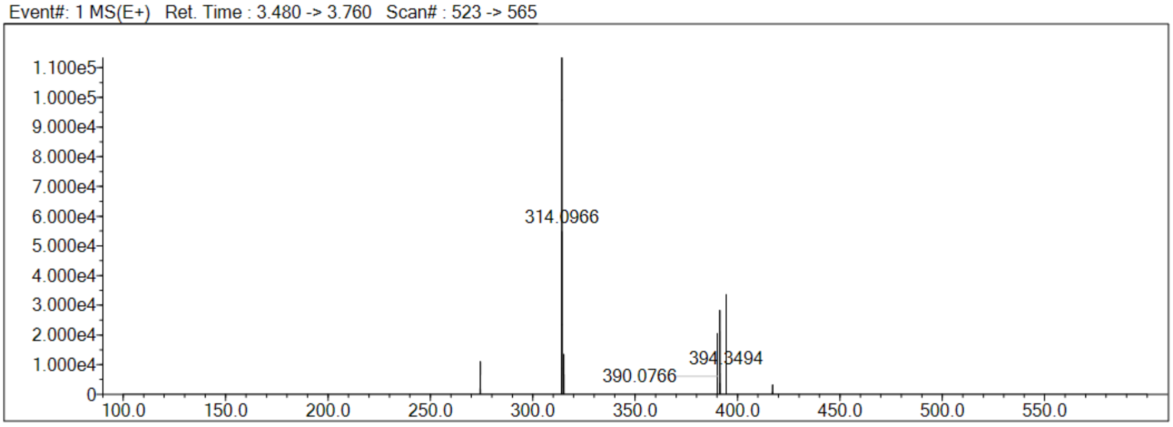

Measured region for 314.0966 m/z

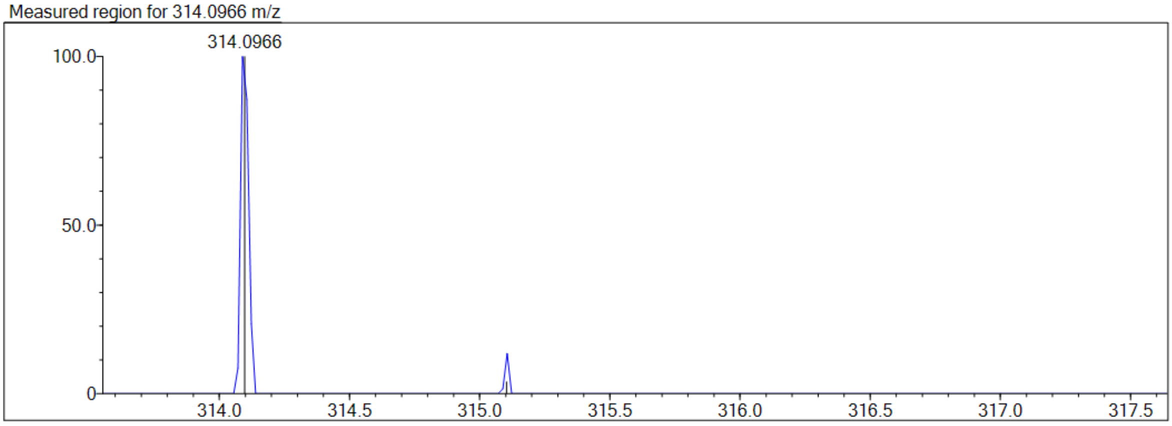

C16 H15 N3 O2 S [M+H]+ : Predicted region for 314.0958 m/z

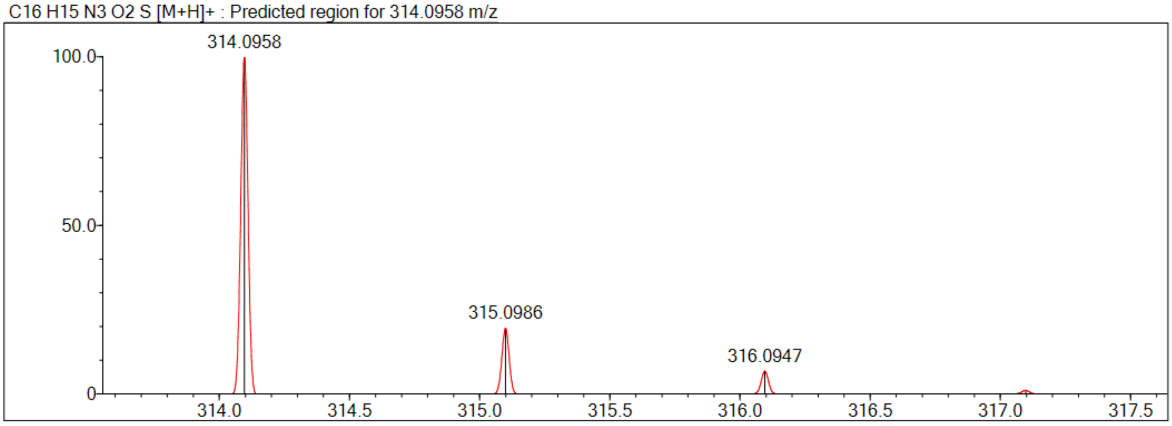

Rank	Score	Formula (M)	Ion	Meas. m/z	Pred. m/z	Df. (mDa)	Df. (ppm)	Iso	DBE
1	0.00	C16 H15 N3 O2 S	[M+H]+	314.0966	314.0958	0.8	2.55	0.00	11.0

Figure S17. Mass spectrum of compound **2g**

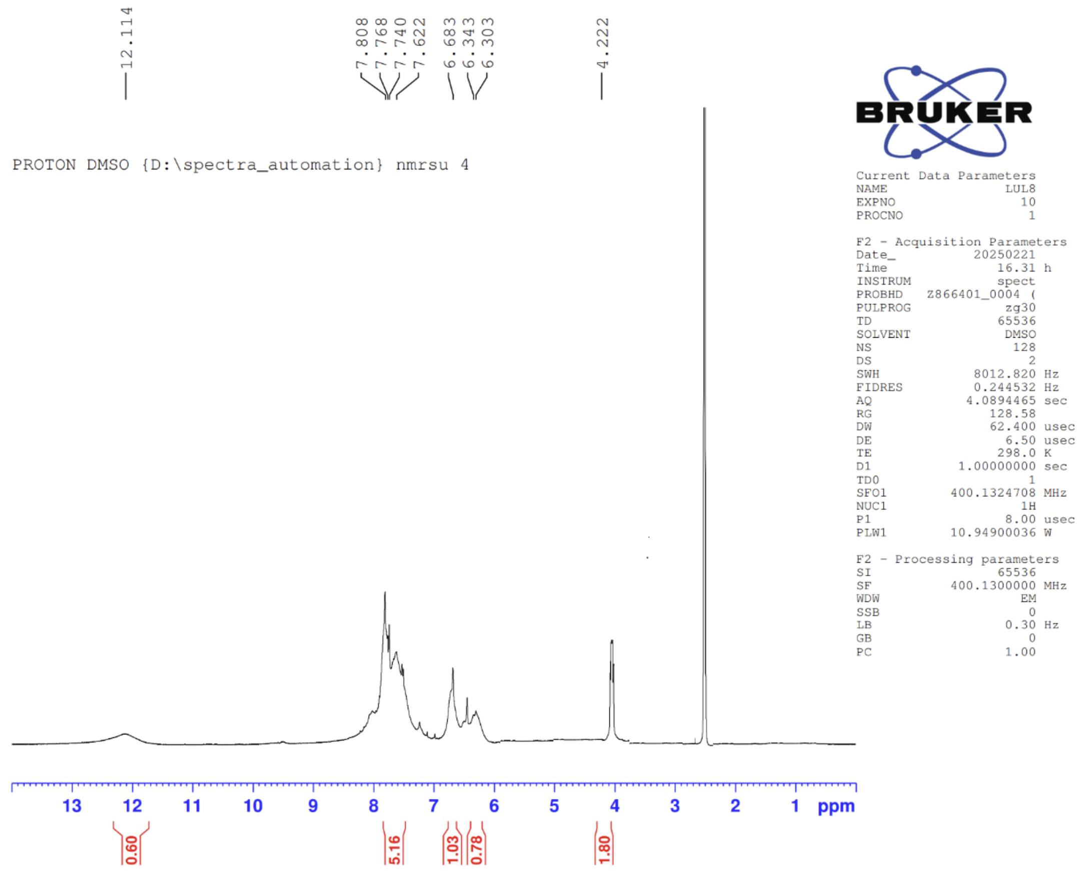

Figure S18. ^{1}H-NMR spectrum of compound **2h**

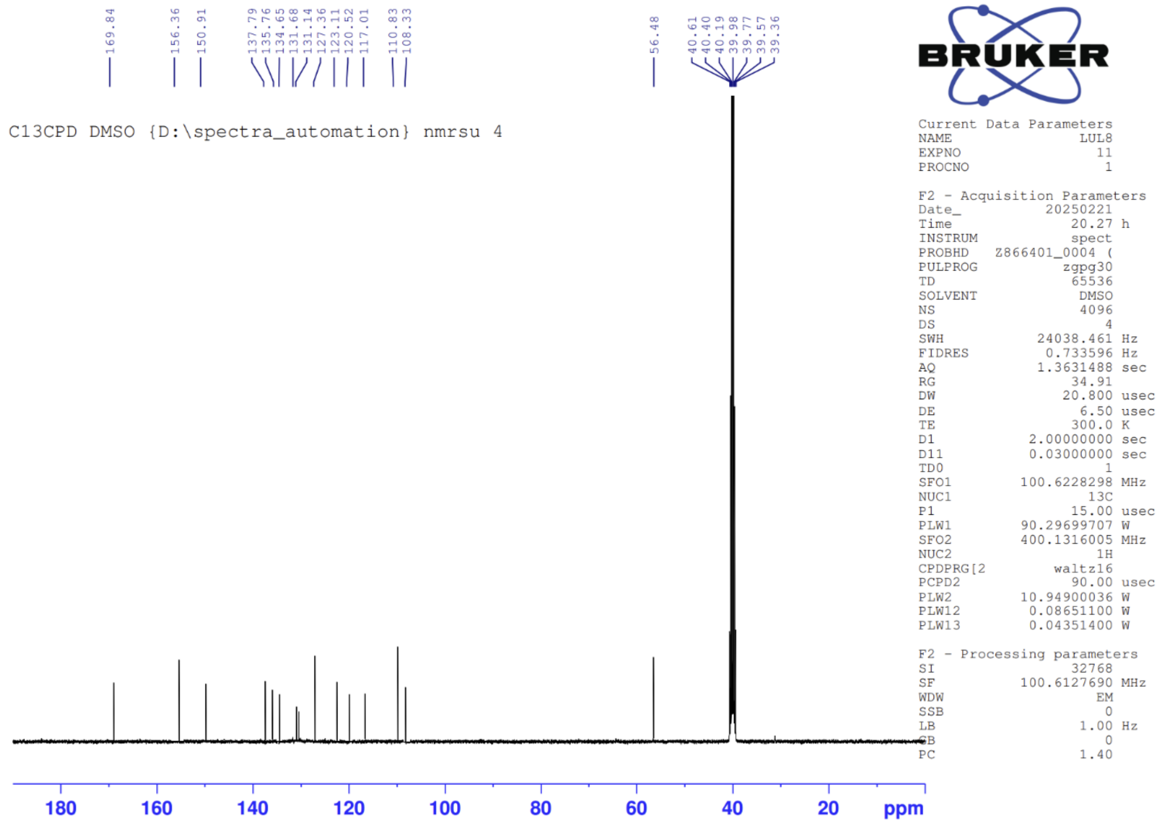

Figure S19. ^{13}C-NMR spectrum of compound **2h**

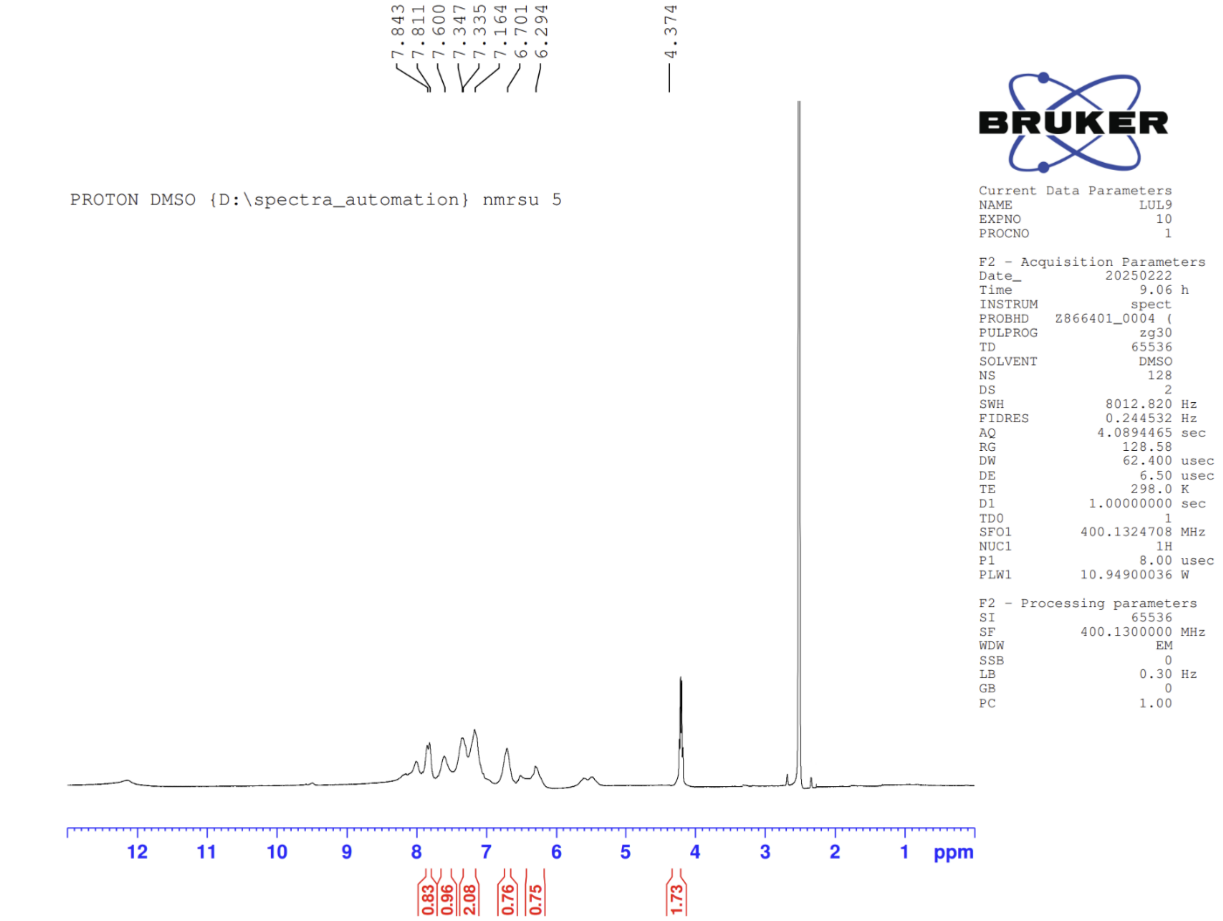

Figure S20. ^{1}H-NMR spectrum of compound **2i**

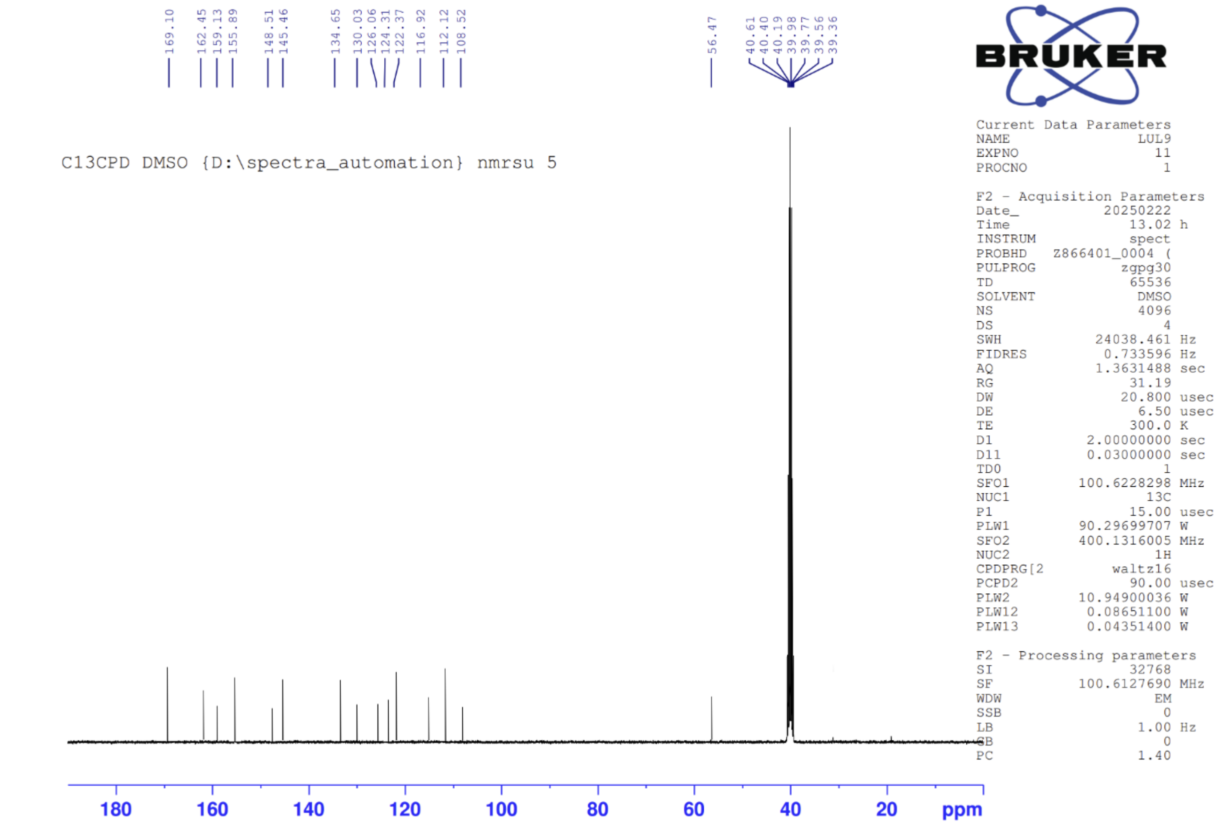

Figure S21. ^{13}C-NMR spectrum of compound **2i**

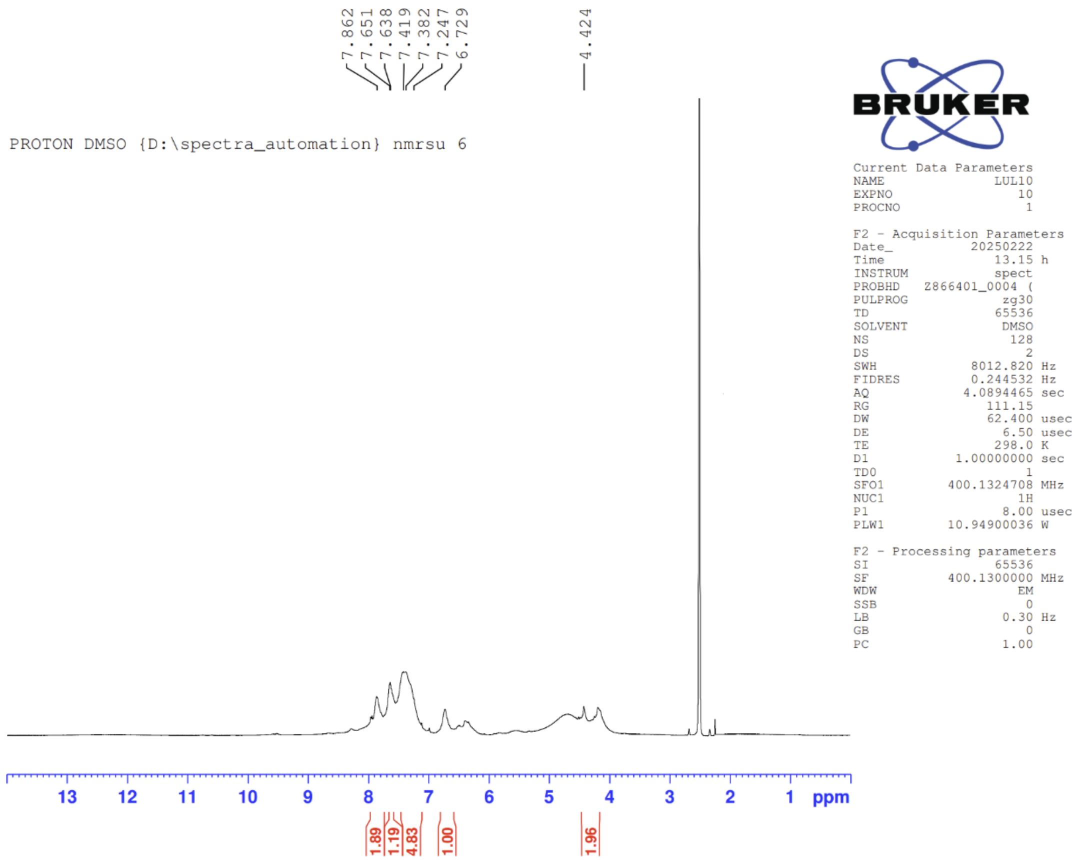

Figure S22. ^{1}H-NMR spectrum of compound **2j**

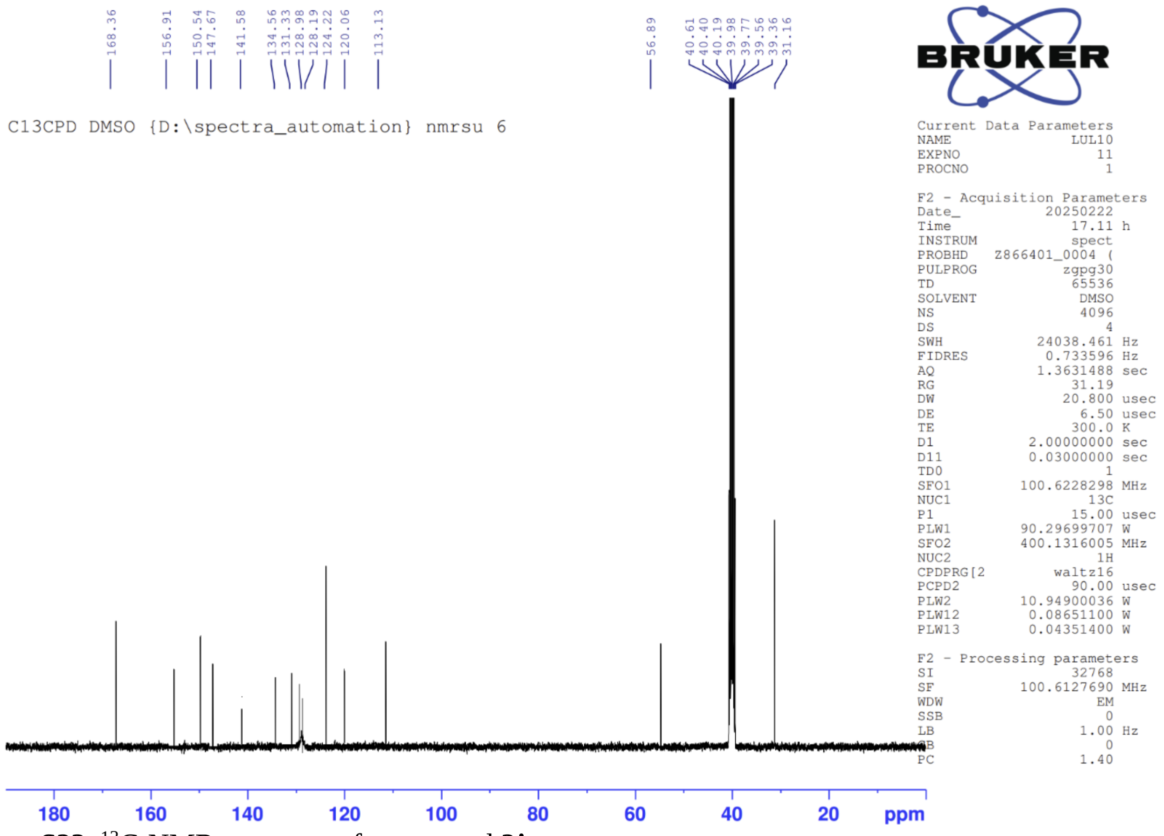

Figure S23. ^{13}C-NMR spectrum of compound **2j**

Data File: C:\LabSolutions\Data\Analiz\uac\LUL-10_146.lcd

Elmt	Val.	Min	Max	Elmt	Val.	Min	Max	Elmt	Val.	Min	Max	Elmt	Val.	Min	Max	Use Adduct
H	1	5	35	O	2	0	5	S	2	1	1	Ru	2	0	0	H
C	4	5	26	F	1	0	0	Cl	1	0	0	Pd	2	0	0	
N	3	0	5	P	3	0	0	Br	1	0	0	I	3	0	0	

Error Margin (ppm): 5
HC Ratio: unlimited
Max Isotopes: 3
MSn Iso RI (%): 10.00

DBE Range: 10.0 - 20.0
Apply N Rule: yes
Isotope RI (%): 1.00
MSn Logic Mode: AND

Electron Ions: both
Use MSn Info: yes
Isotope Res: 9000
Max Results: 500

Event#: 1 MS(E+) Ret. Time : 2.827 -> 3.133 Scan# : 425 -> 471

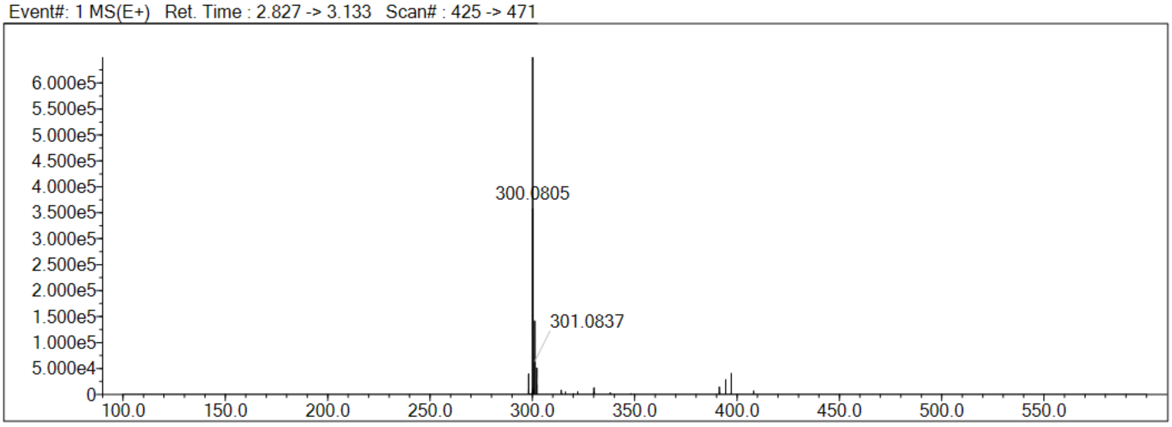

Measured region for 300.0805 m/z

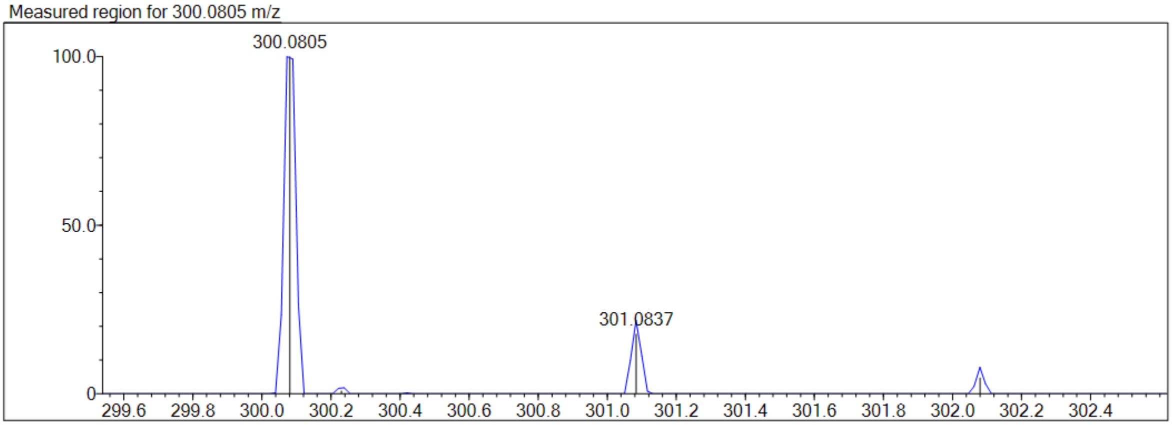

C15 H13 N3 O2 S [M+H]+ : Predicted region for 300.0801 m/z

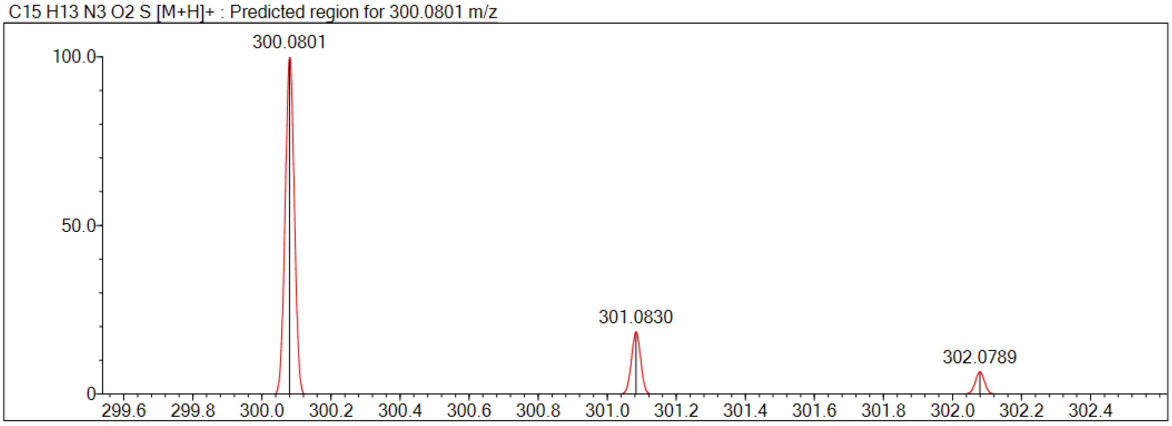

Rank	Score	Formula (M)	Ion	Meas. m/z	Pred. m/z	Df. (mDa)	Df. (ppm)	Iso	DBE
1	97.73	C15 H13 N3 O2 S	[M+H]+	300.0805	300.0801	0.4	1.33	98.54	11.0

Figure S24. Mass spectrum of compound **2j**

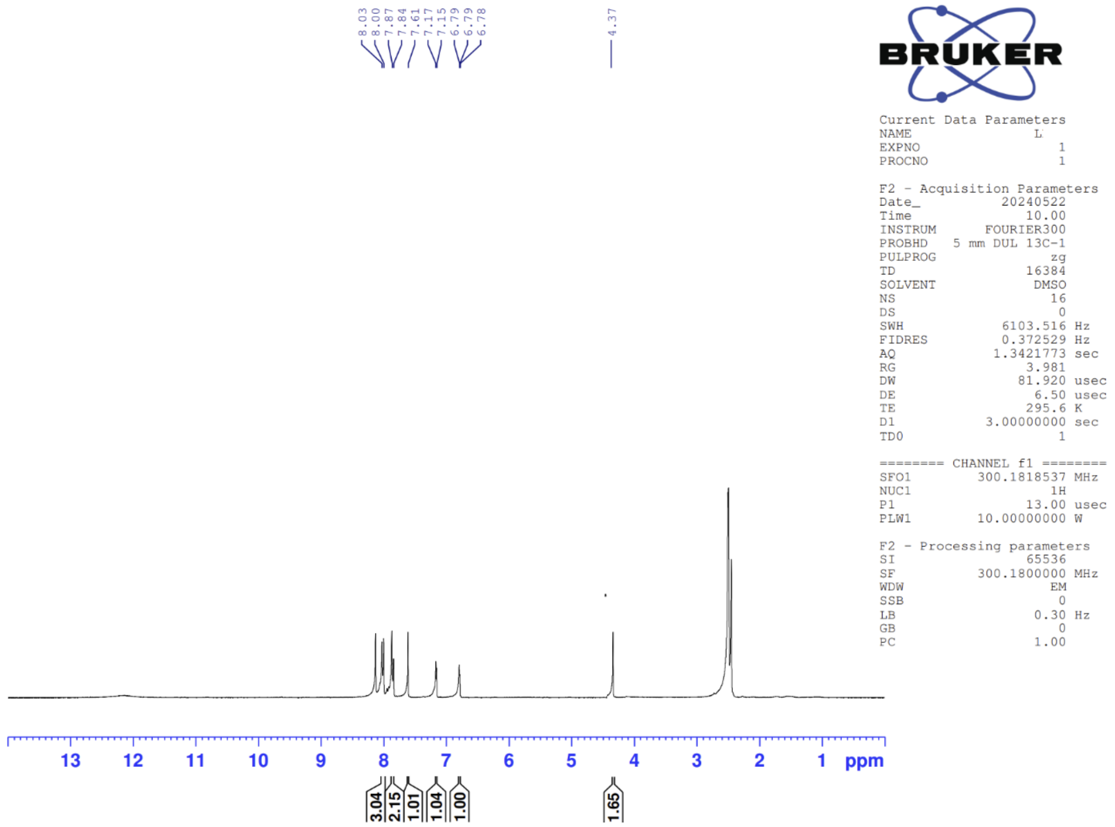

Figure S25. ^{1}H-NMR spectrum of compound **2k**

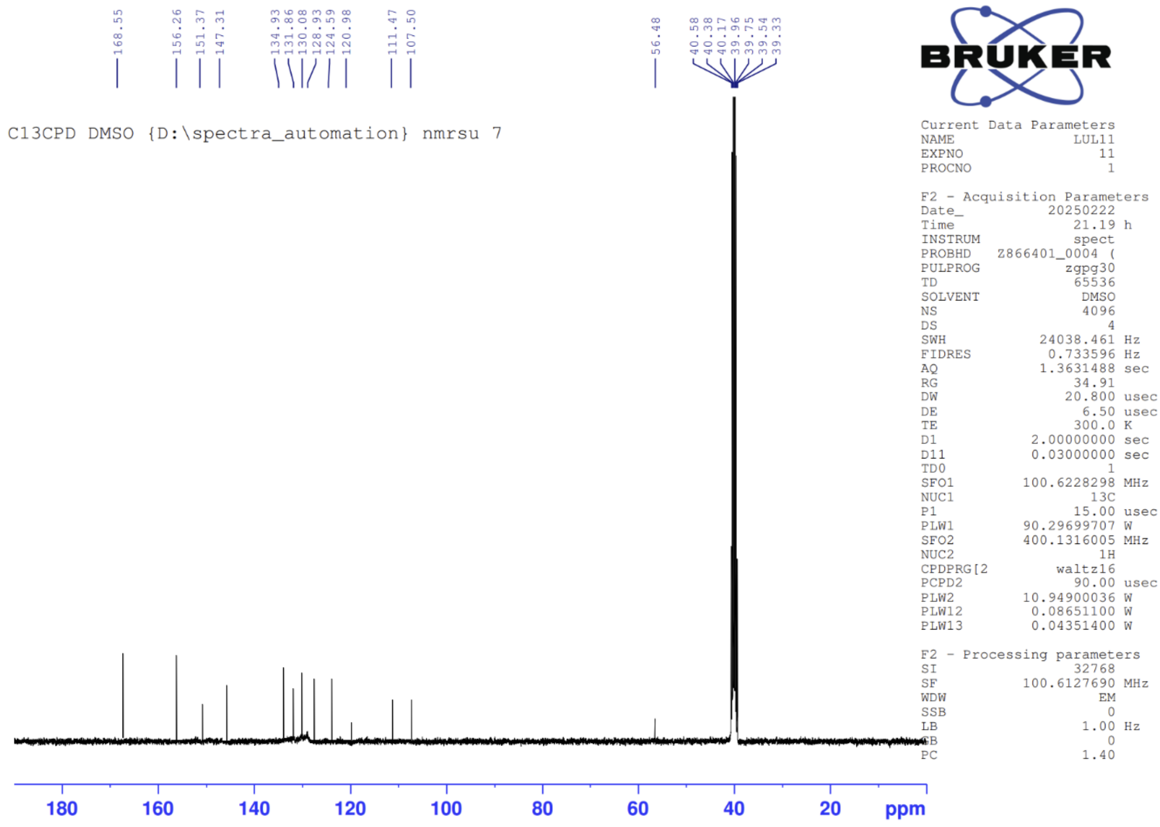

Figure S26. ^{13}C-NMR spectrum of compound **2k**

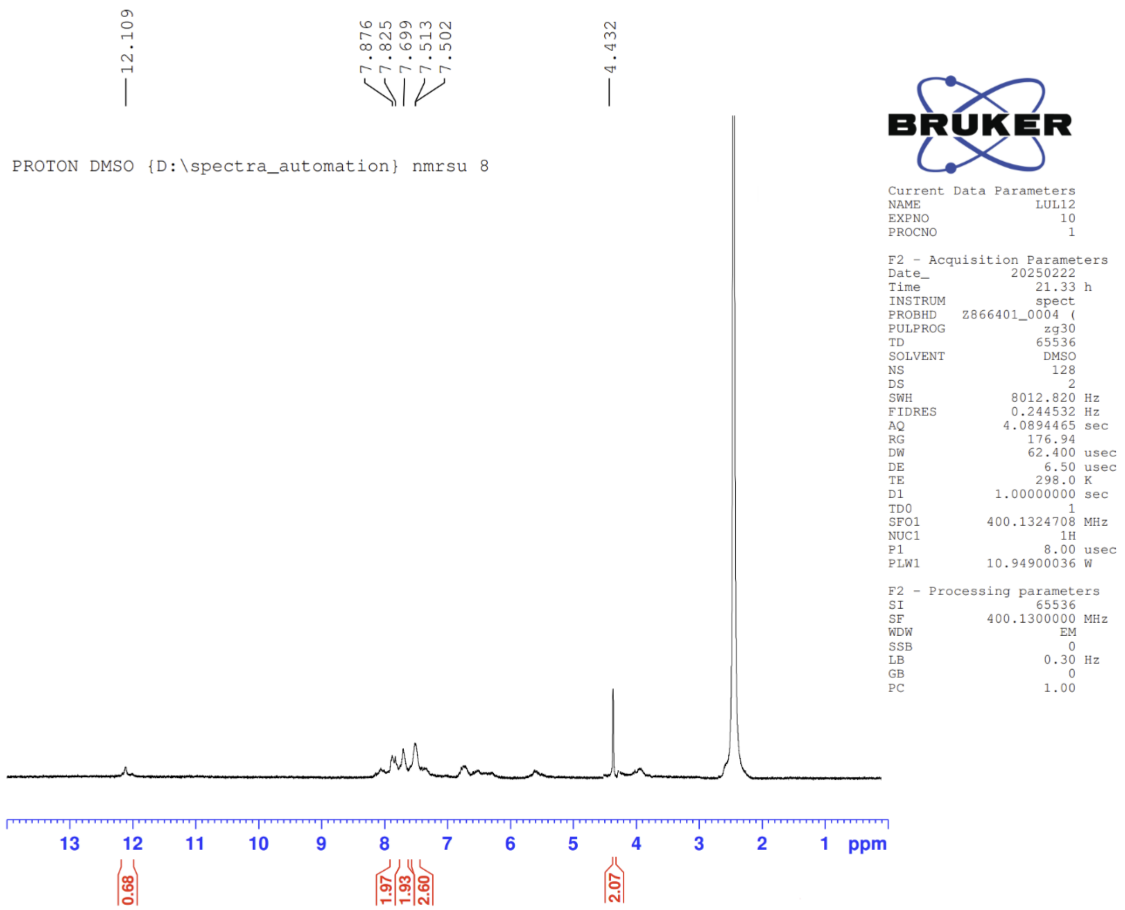

Figure S27. ^{1}H-NMR spectrum of compound **2l**

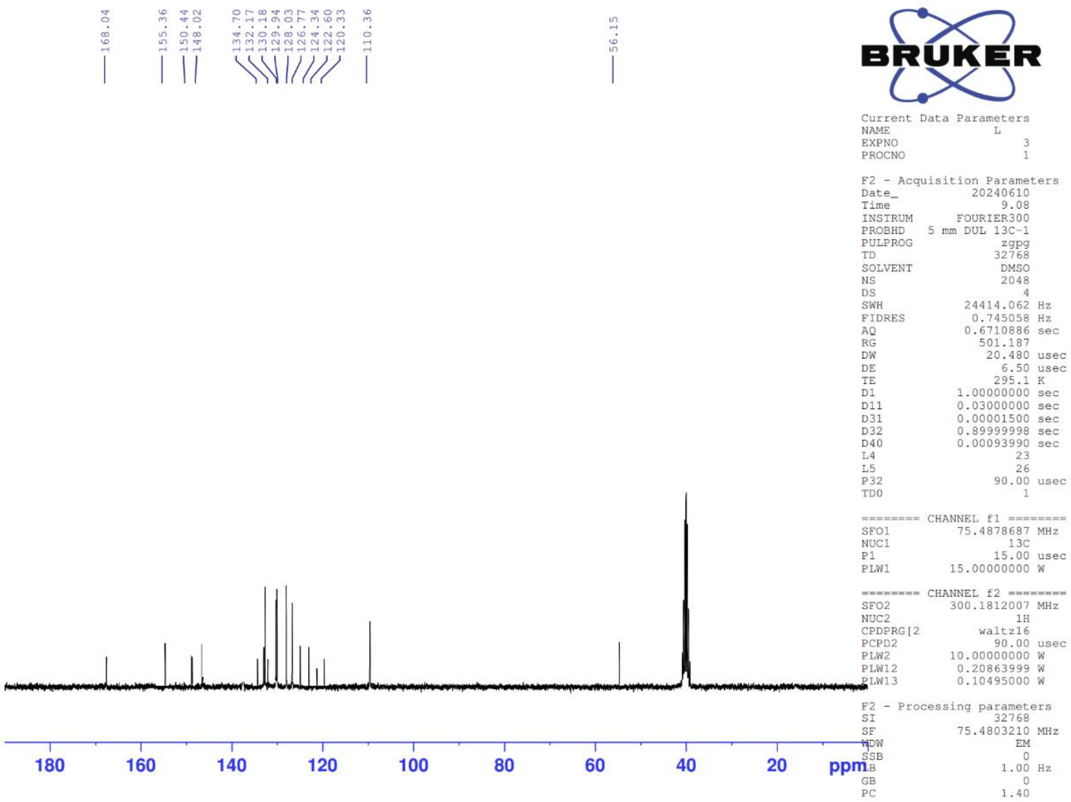

Figure S28. ^{13}C-NMR spectrum of compound **2l**

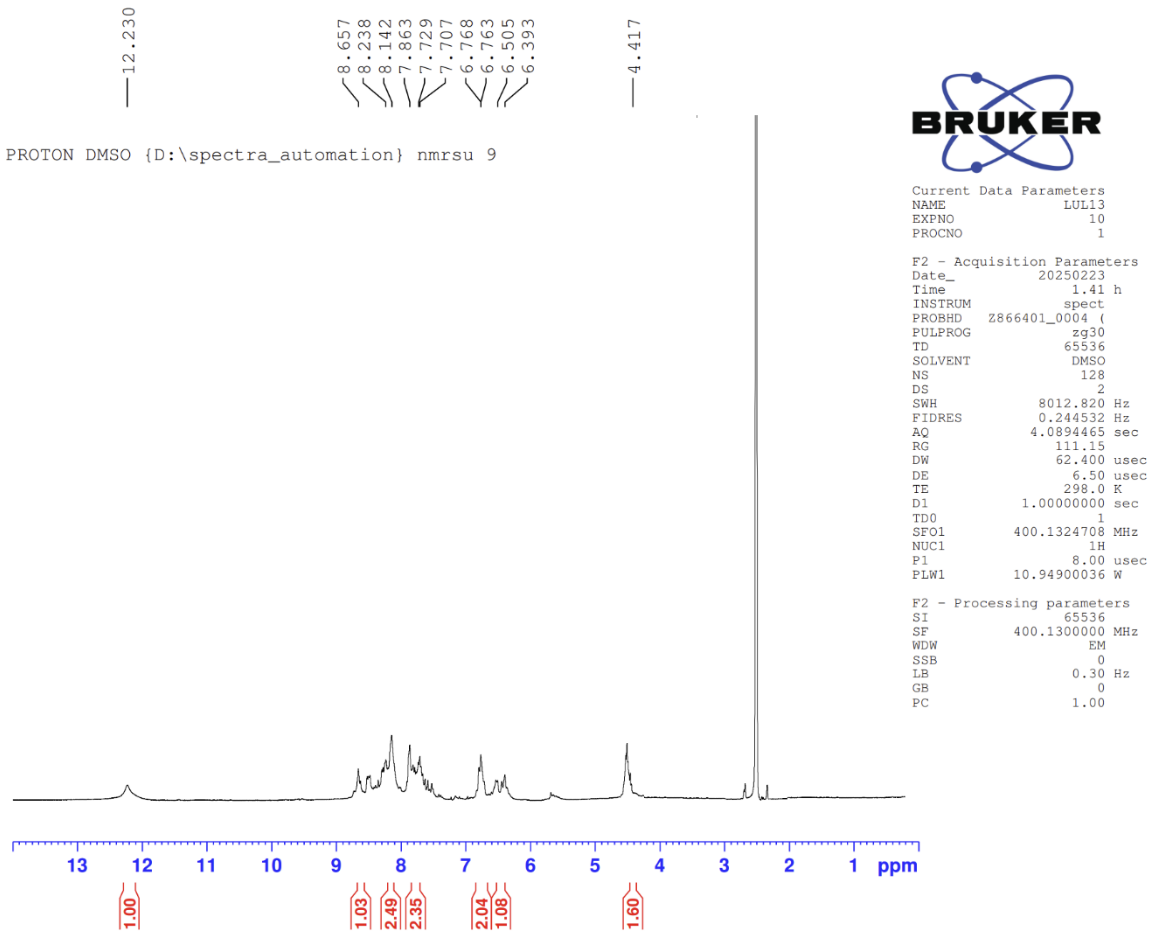

Figure S29. ^{1}H-NMR spectrum of compound **2m**

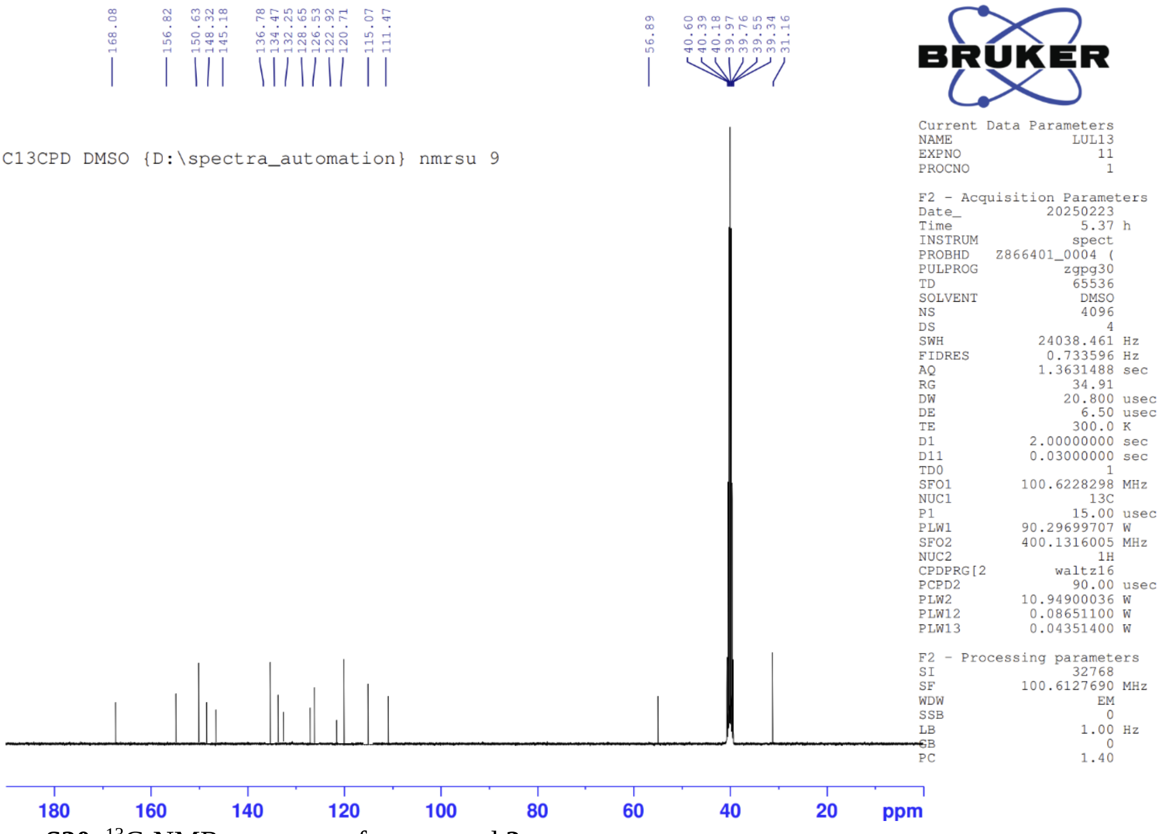

Figure S30. ^{13}C-NMR spectrum of compound **2m**
